# Efficacy of acupuncture therapy plus related rehabilitation therapy for post-stroke urinary incontinence: a systematic review and meta-analysis

**DOI:** 10.3389/fneur.2025.1575970

**Published:** 2025-05-08

**Authors:** Zifeng Dai, Yuting Wang, Yuzheng Du, Linru Hou, Yufen Li, Kaixuan Ma, Qinfeng Yan, Jian Wen, Xinlei Dong, Xiaolin Chen, Lili Zhang

**Affiliations:** ^1^First Teaching Hospital of Tianjin University of Traditional Chinese Medicine, Tianjin, China; ^2^National Clinical Research Center for Chinese Medicine Acupuncture and Moxibustion, Tianjin, China

**Keywords:** prick, rehabilitation therapy, stroke, urinary incontinence, meta-analysis

## Abstract

**Introduction:**

Researchers have increasingly focused on the efficacy of acupuncture therapy (AT) combine with rehabilitation therapy (RT) for post-stroke urinary incontinence (PSUI). This study aims to fully assess the efficacy of AT plus related RT in treating PSUI.

**Methods:**

We systematically searched eight databases from their inception to March 2025 for randomized controlled trials (RCTs) evaluating AT plus related RT for PSUI. Stata 18.0 was utilized for the meta-analyses.

**Results:**

Thirty-six studies involving 2,796 subjects were included, with AT plus related RT performed in the treatment group. The total effective rate of AT plus RT was significantly higher than that of RT or AT alone [RR = 1.23, 95% CI (1.19, 1.28), *p* < 0.001]. AT plus RT was also superior to related RT or related AT in improving maximum bladder capacity [WMD = 44.93, 95% CI (32.00, 57.87), *p* < 0.001]; increasing maximum urinary flow rate [WMD = 2.64, 95% CI (1.27, 4.01), *p* < 0.001], mean urine output per time [WMD = 44.30, 95% CI (20.31, 68.29), *p* < 0.001], and pelvic floor muscle strength (including fast [WMD = 2.64, 95% CI (1.04, 4.25), *p* = 0.001], slow [WMD = 6.09, 95% CI (3.44, 8.75), *p* < 0.001], and complex muscle fibers [WMD = 5.46, 95% CI (3.60, 7.32), *p* < 0.001]); and reducing the residual urine volume [WMD = −20.84, 95% CI (−27.53, −14.14), *p* = 0.001], maximal detrusor pressure [WMD = −10.6, 95% CI (−12.72, −8.55), *p* = 0.001], frequency of 24-h UI [WMD = −1.40, 95% CI (−1.92, −0.88), *p* < 0.001], and frequency of 24-h urination [WMD = −3.76, 95% CI (−4.87, −2.66), *p* < 0.001]. Moreover, AT plus RT significantly reduced scores on the International Consultation on Incontinence Questionnaire-Short Form (ICIQ-SF) [WMD = −2.40, 95% CI (−2.93, −1.83), *p* < 0.001]. While reductions were also observed in the quality of life (QOL) score [WMD = −0.72, 95% CI (−1.64, 0.20), *p* = 0.127] and the National Institutes of Health Stroke Scale (NIHSS) score [WMD = −3.51, 95% CI (−8.20, 1.18), *p* = 0.143], these did not reach statistical significance. Additionally, AT plus RT significantly increased the Incontinence Quality of Life Scale (I-QOL) score [WMD = 11.71, 95% CI (8.10, 15.33), *p* < 0.001] and the Barthel index (BI) score [WMD = 6.92, 95% CI (−0.22, 14.05), *p* = 0.058].

**Discussion:**

AT plus RT outperforms related RT or related AT in improving clinical efficacy and bladder function in PSUI patients. However, the number of included studies on AT plus RT remains limited, highlighting the need for more high-quality RCTs are needed to validate the findings.

**Systematic review registration:**

https://www.crd.york.ac.uk/prospero/, identifier [CRD42024588520].

## 1 Introduction

As the second major contributor to global disability and death, stroke causes approximately 5.5 million deaths annually (1), posing an increasing socioeconomic burden (2). Over the past decades, post-stroke urinary incontinence (PSUI) has been recognized as a major post-stroke sequela (3). The prevalence of PSUI was 38% in a meta-analysis involving 7,327 stroke patients (4). PSUI may further lead to urinary system infections and pressure injuries (5), increase the incidence of decubitus ulcers, urinary tract infections, and dermatitis (6–10), and induce negative social mentality (e.g., self-abasement, self-importance, anxiety, embarrassment, depression, or social isolation). In addition, PSUI imposes a burden on caregivers (11), thus affecting the prognosis and survival of stroke patients. Therefore, PSUI rehabilitation and treatment are particularly important for helping patients return to normal both physically and psychologically and easing the socioeconomic burden.

In addition to pharmacological and surgical treatments, rehabilitation therapy (RT) can also be used to treat PSUI. RT primarily targets the corresponding muscles or innervating nerves of the pelvic floor or bladder or the corresponding regions of the central nervous system (12, 13). However, they fail to fully improve the quality of life (QOL) or physical functioning of PSUI patients. For example, pelvic floor muscle training (PFMT) does not significantly ameliorate emotional wellbeing or activities of daily living in patients with post-stroke lower urinary tract syndrome as compared to controls and exerts no positive influence on the nocturia-related QOL (14). Compared to bladder training (BT) alone, pelvic floor muscle electrical stimulation (PFMES) combined with BT does not lead to improvements in the amount of urine leakage and the Bristol Female Urinary Symptoms Questionnaire (BFUSQ) and International Consultation on Incontinence Questionnaire-Short Form (ICIQ-SF) scores in PSUI patients early in treatment (15).

Acupuncture therapy (AT), characterized by simple operation, small side effects, and high acceptance, has been recommended by the National Institutes of Health as an adjunct to stroke rehabilitation (16). Acupuncture (A), electroacupuncture (EA), warm acupuncture (WA), and ear acupuncture are the main techniques of traditional AT, and their advantages over other treatments in PSUI rehabilitation have been revealed. For example, EA can improve the voiding diary scores, patient satisfaction, and bladder capacity in PSUI patients (17) and reduce the severity and symptom scores of incontinence (18). Moreover, a growing body of research suggests that AT plus RT may achieve a better effect on PSUI rehabilitation than RT or AT alone. AT plus related RT is also superior to either RT (19, 20) or AT (21) alone in decreasing the clinical ineffective rate, ameliorating clinical symptoms, enhancing bladder function, and improving the patient’s ability to voluntarily urinate and QOL.

Therefore, this systematic review and meta-analysis intends to reveal the effect of AT plus related RT vs. related RT or related AT alone on PSUI, thereby offering evidence for clinical practice.

## 2 Methods

This study was conducted following the Preferred Reporting Items for Systematic Reviews and Meta-Analyses (PRISMA 2020) statement (22), and its protocol was registered with PROSPERO (CRD42024588520).

### 2.1 Search strategy

We searched the databases of Web of Science, Embase, PubMed, Cochrane Library, CNKI, Wanfang Data, VIP, and SinoMed from inception to March 2025. The search term was composed of medical subject headings and free words, including “acupuncture,” “stroke,” “urinary incontinence,” and “randomized”. The search strategy is detailed in Supplementary material.

### 2.2 Eligibility criteria

The inclusion criteria were as follows: (1) Population: PSUI patients, with no restriction on the stroke type, gender, or ethnicity; (2) Interventions: AT (A, EA, WA, ear acupuncture, head acupuncture, and abdominal acupuncture) plus related RT [PFMT, PFMES, pelvic floor muscle magnetic stimulation (PFMMS), BT, transcranial direct current stimulation (tDCS), and biofeedback therapy (BFT)]; BT included pelvic floor muscle massage, water intake planning, urination habit training, and intermittent catheterization; (3) Comparisons: related RT alone or a combination of multiple rehabilitation approaches or AT alone; (4) Outcomes: effective rate, maximum bladder capacity, residual urine volume (RUV), ICIQ-SF score, maximum urinary flow rate (Qmax), QOL score, maximal detrusor pressure (MDP), Incontinence Quality of Life Scale (I-QOL) score, urethral closure pressure, Barthel index (BI) score, National Institutes of Health Stroke Scale (NIHSS) score, frequency of 24-h UI, mean urine output per time, and pelvic floor muscle strength; (5) Study design: RCTs.

The exclusion criteria were as follows: (1) the interventions included moxibustion; (2) conference minutes, comments, letters to the editor, case reports, and reviews; (3) studies that were not available in full text, did not describe the intervention process in detail, or lacked complete data; (4) duplicate publications with the same populations included and highly similar outcomes.

### 2.3 Study screening

Two reviewers (HL and LY) independently conducted study screening. After duplicate removal, the title and abstract were reviewed to initially include the relevant studies. Then, the full text was examined to finally include the eligible studies. Discrepancies were settled by a third reviewer (ZL).

### 2.4 Quality assessment

The two reviewers (HL and LY) independently assessed the quality of the included studies using the Cochrane risk-of-bias tool 2.0 (23). Risk of bias (RoB) was assessed in five domains: bias arising from the randomization process (D1, from randomization, allocation concealment, and baseline differences), bias due to deviations from intended interventions (D2, from intervention perception, intervention, and effect), bias due to missing outcome data (D3), bias in the measurement of the outcome (D4, from measurement methodology, intergroup consistency, and knowledge of the allocated interventions by assessors), and bias in selection of the reported result (D5, based on the pre-specified analysis approach and the likelihood of outcome selection). Each study was categorized as “low risk,” “some concerns,” or “high risk.” The overall RoB was considered low if all assessments were “low risk”; otherwise, it was categorized as “some concerns” or “high risk.” Discrepancies were settled by a third reviewer (ZL).

### 2.5 Data extraction

Two reviewers (HL and LY) extracted data using a spreadsheet pre-formulated according to the Cochrane Handbook: authors, year of publication, demographic characteristics, interventions, and outcomes. The outcomes included the efficacy evaluation (total effective rate), objective indicators of bladder function (maximum bladder capacity, Qmax, RUV, MDP, frequency of 24-h UI, frequency of 24-h urination, mean urine output per time, and pelvic floor muscle strength), and scores from subjective scales of stroke severity, urinary incontinence symptoms, and QOL (ICIQ-SF, BI, QOL, NIHSS, and I-QOL scores). Discrepancies were resolved by consulting a third reviewer (ZL).

### 2.6 Data analysis

Meta-analyses were performed using Stata 18.0. First, Cochran’s Q test and *I*^2^ statistic were used for heterogeneity tests. A fixed-effects model was adopted when heterogeneity was acceptable (*I*^2^ < 50%, *p* > 0.05); otherwise, a random-effects model was adopted due to significant heterogeneity (*I*^2^ ≥ 50%, *p* < 0.05). If data permitted, subgroup analyses were conducted based on the treatment course, patient age, stroke type, RT, and AT to explore the sources of heterogeneity. Dichotomous variables were presented as risk ratios (RRs) and continuous variables as mean deviations (MDs), both with 95% confidence intervals (CIs) calculated. A *p*-value of <0.05 was deemed statistically significant. If the number of included studies on one outcome metric was >10, we assessed publication bias (24) by Egger’s tests and funnel plots. A *p*-value of <0.05 (Egger’s test) was deemed to be due to the presence of publication bias (25–27), and then, whether publication bias had a great influence on the results was evaluated using the trim-and-fill method. We also investigated whether the results were robust using sensitivity analyses.

## 3 Results

### 3.1 Search results and basic characteristics

**Figure 1:**
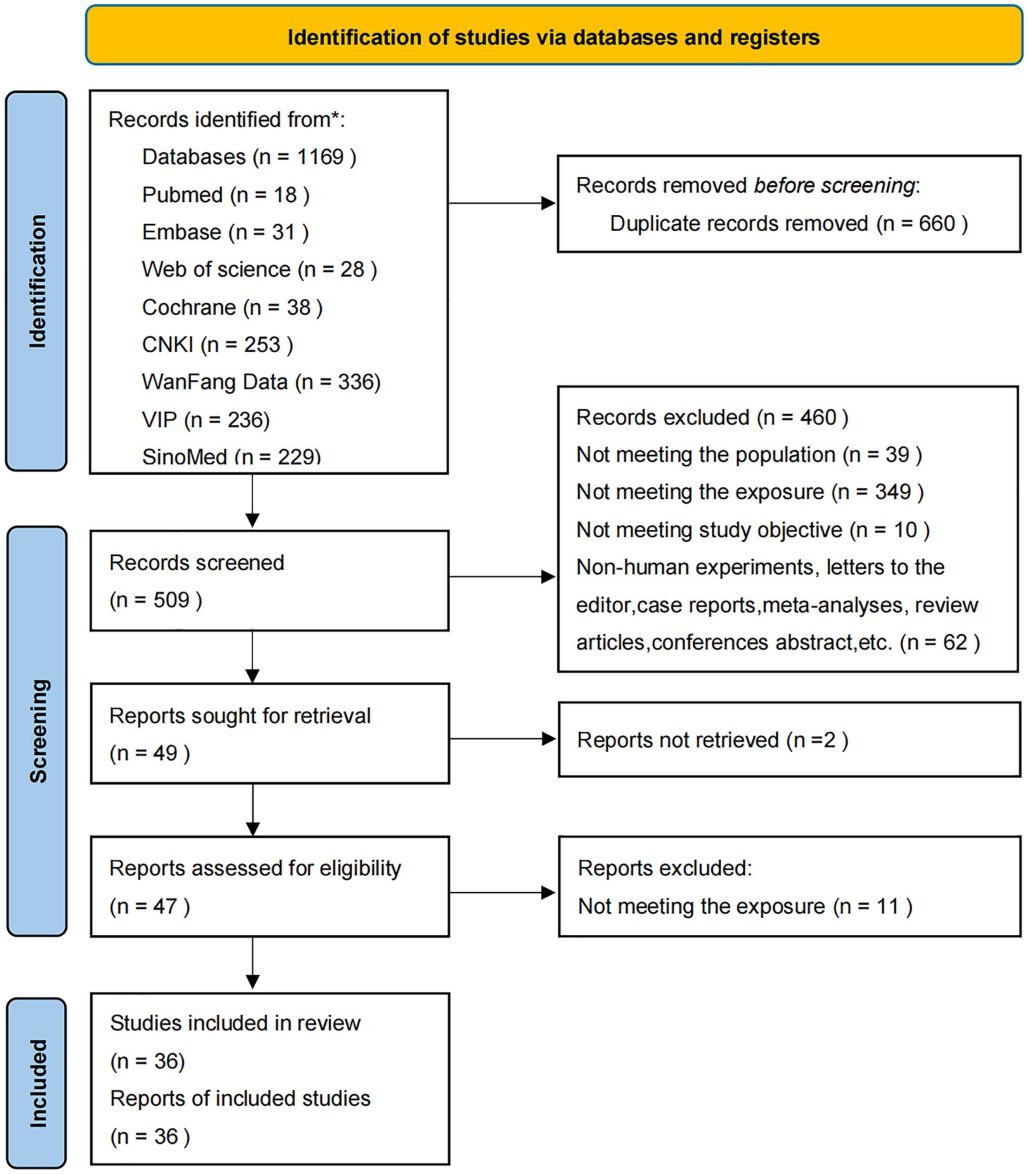
Flowchart of study screening.

Initially, 1,169 Chinese-and English-language studies were retrieved, of which 660 duplicates were excluded. After title and abstract reviewing, 460 studies were excluded. Then, the full text of the remaining 49 studies was assessed according to the eligibility criteria. Finally, 36 studies (21, 28–62) were included (Figure 1), involving 2,796 subjects with a mean age of 58.63 years. These comprised 1,397 cases given AT plus related RT, 1,164 cases given related RT alone, and 235 cases given AT alone (Table 1). A total of 2,046 subjects with stroke were in the acute or subacute phase, and 146 were in the chronic phase; and the stroke stage was not reported for the remaining 604 subjects (63).

**Table 1:** Basic study characteristics.

Author and Year	Country	Types of Strokes	Sample Size experimental group per control group	Age mean ± SD, or range	Intervention (experimental group)	Intervention (control group)	Treatment cycle (experimental group)	Treatment cycle (control group)
Cai et al. (2024) (24)	China	CI	54 per 54	39.5 ± 7.98	WA + PFMES+BT	PFMES+BT	48d (15 times per 16d)	90d (1 time per d)
Wang et al. (2024) (4)	China	S	43 per 43	67.58 ± 5.71	A + BT	BT	28d (6 times per 7d)	28d (1 time per d)
Qu et al. (2024) (31)	China	S	51 per 51	60.48 ± 5.15	EA + PFMES+BT + PFMT	BT + PFMT	28d (1 time per d)	28d (1 time per d)
Han (2024) (28)	China	S	36 per 36	59.42 ± 7.21	A + BT + PFMT+PFMES	BT + PFMT+PFMES	21d (5 times per 7d)	21d (5 times per 7d)
Liu et al. (2024) (21)	China	S	40 per 40	63.04 ± 7.67	A + PFMMS	A	14d (1 time per d)	14d (1 time per d)
Duan et al., 2023 (34)	China	CI	37 per 37	50.47 ± 9.23	A + PFMES	PFMES	14d (5 times per 7d)	30d (2 times per d)
Wei et al. (2023) (32)	China	S	42 per 42	54.35 ± 6.91	A + BT + TDCS	BT + TDCS	56d (5 times per 7d)	56d (5 times per 7d)
Wang (2023) (33)	China	S	36 per 36	47.51 ± 11.42	A + PFMMS+PFMT	PFMMS+PFMT	28d (6 times per 7d)	28d (1 time per 2d)
Pan et al. (2023) (35)	China	S	30 per 30	62.86 ± 10.34	A + PFMMS	PFMMS	28d (5 times per 7d)	28d (5 times per 7d)
Nie et al. (2022) (36)	China	CH	21 per 21	54.1 ± 9.94	EA + BT	BT	84d (1 time per d)	84d (1 time per d)
Wang et al. (2022) (37)	China	S	36 per 36	57.7 ± 15	A + PFMES	PFMES	21d (6 times per 7d)	21d (6 times per 7d)
Zhang et al. (2022) (38)	China	S	45 per 45	46.31 ± 3.83	A + PFMT	PFMT	39d (10 times per 13d)	30d (1 time per d)
Zhuang et al. (2022) (39)	China	S	25 per 25	53.22 ± 8.62	A + TDCS	TDCS	28d (5 times per 7d)	28d (5 times per 7d)
Liu (2022) (40)	China	S	37 per 37	62.5 ± 9.49	PFMES+PFMES	PFMES	21d (6 times per 7d)	21d (6 times per 7d)
Wu et al. (2021) (42)	China	S	30 per 30	60.5 ± 8.01	A + PFMT	PFMT	42d (6 times per 7d)	42d (1 time per d)
Chen (2021) (41)	China	S	25 per 26	51.06 ± 10.02	A + TDCS	A	28d (5 times per 7d)	28d (5 times per 7d)
Qiu (2020) (44)	China	S	55 per 54	41–77	WA + PFMT	PFMT	24d (5 times per 6d)	24d (3 times per d)
Li, 2020 (46)	China	S	42 per 42	68.03 ± 4.84	WA + BFT	BFT	28d (5 times per 7d)	20d (2 times per d)
Gu (2020) (43)	China	S	30 per 30	56.4 ± 11.33	A + PFMT	PFMT	21d (6 times per 7d)	21d (6 times per 7d)
Yang et al. (2020) (45)	China	S	50 per 50	62.7 ± 9.02	A + EA + PFMT+BT	PFMT+BT	28d (1 time per d)	28d (1 time per d)
Wu (2020) (48)	China	S	22 per 23	67.93 ± 9.29	A + BT	BT	28d (5 times per 7d)	28d (1 time per d)
Hua et al. (2020) (49)	China	CI	49 per 48	61.01 ± 8.02	A + PFMT+PFMES	PFMT+PFMES	28d (5 times per 7d)	28d (5 times per 7d)
Zhu et al. (2020) (47)	China	S	44 per 44	63.61 ± 2.56	A + BT	A	60d (1 time per d)	60d (1 time per d)
Xing et al. (2019) (50)	China	S	41 per 41	41.12 ± 4.47	EA + PFMT	PFMT	14d (6 times per 7d)	14d (1 time per d)
Li and Qu (2019) (51)	China	CI	48 per 48	45.71 ± 5.35	WA + PFMES+BT + PFMT	PFMES+BT + PFMT	63d (4 times per 7d)	63d (4 times per 7d)
Wu (2019) (52)	China	S	42 per 42	64.47 ± 9.1	A + PFMT+BT	PFMT+BT	28d (1 time per d)	28d (1 time per d)
Ta (2019) (53)	China	S	32 per 32	64.96 ± 6.64	A + PFMT	PFMT	14d (5 times per 7d)	14d (1 time per d)
Dong and Shi (2019) (54)	China	CI	56 per 56	74.3 ± 10.02	A + PFMT	PFMT	28d (1 time per d)	28d (1 time per d)
Zhao et al. (2016) (55)	China	CI	72 per 72	60.83 ± 5.25	EA + PFMT	PFMT	84d (3 times per 7d)	84d (3 times per d)
Gu et al. (2015) (43)	China	CI	59 per 59	66.1 ± 10.78	A + PFMT	PFMT	28d (1 time per d)	28d (1 time per 2d)
Wang et al. (2014) (57)	China	S	15 per 15	55 ± 1.02	A + PFMES	PFMES	28d (6 times per 7d)	28d (1 time per 2d)
Li and Cheng (2014) (59)	China	S	30 per 30	63.5 ± 7.3	A + BT + PFMT+PFMES	A	30d (1 time per d)	30d (1 time per d)
Wang et al. (2014) (58)	China	S	30 per 30	57.8 ± 0.39	A + PFMES	A	14d (6 times per 7d)	14d (6 times per 7d)
Zhang et al. (2013) (60)	China	S	30 per 30	64.94 ± 5.71	A + PFMT	A	34d (14 times per 17d)	34d (14 times per 17d)
Feng and Bai (2011) (61)	China	NA	30 per 29	60–78	EA + BT	BT	28d (5 times per 7d)	28d (5 times per 7d)
Wang et al. (2009) (62)	China	S	32 per 35	41–68	A + PFMES	A	30d (1 time per d)	30d (1 time per d)

CI, cerebral infarction; S, stroke (cerebral infarction and hemorrhage); CH, cerebral hemorrhage; A, acupuncture; WA, warm acupuncture; EA, electroacupuncture; PFMES, pelvic floor muscle electrical stimulation; TDCS, transcranial direct current stimulation; PFMMS, pelvic floor muscle magnetic stimulation; PFMT, pelvic floor muscle training; BFT, biofeedback therapy; BT, bladder training.

### 3.2 Quality assessment

**Figure 2:**
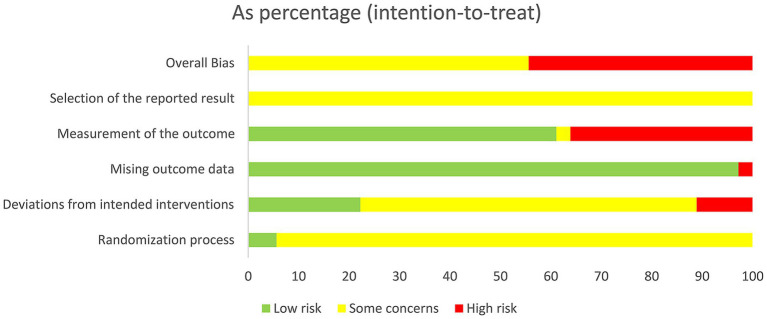
Percentage of risk-of-bias (RoB).

**Figure 3:**
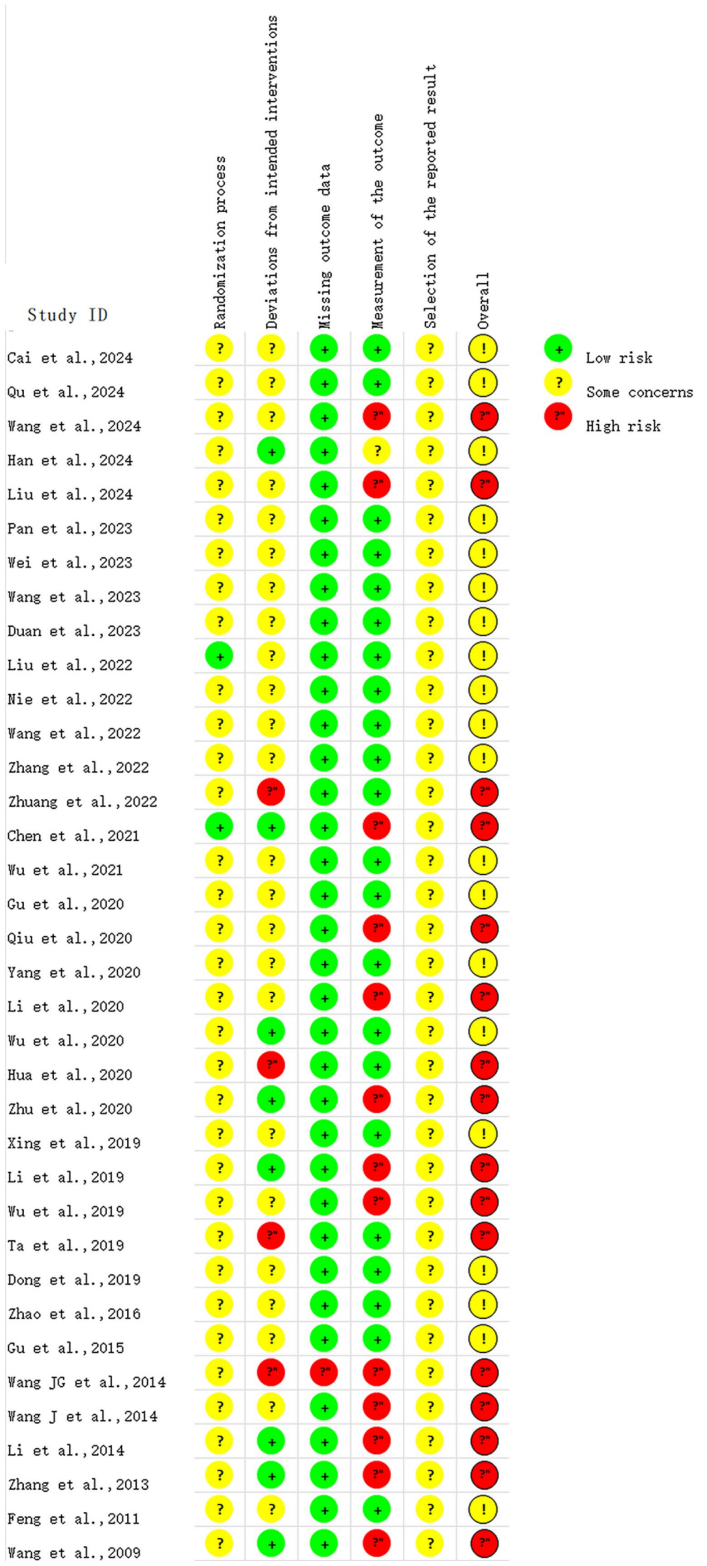
RoB summary.

It was found that using the Cochrane risk-of-bias tool 2.0 (23), the following assessments were made: in D1, 2 studies were rated as “low risk” and 34 as “some concerns”; in D2, 8 studies were rated as “low risk,” 24 as “some concerns,” and 4 as “high risk”; in D3, 35 studies were rated as “low risk” and 1 as “high risk”; in D4, 22 studies were rated as “low risk,” 1 as “some concerns,” and 13 as “high risk”; in D5, all studies were rated as “some concerns.” The overall RoB was “some concerns” in 20 studies and “high risk” in 16 studies. The pooled results are displayed in Figure 2, and the assessment results of individual studies are displayed in Figure 3.

### 3.3 Meta-analysis results

#### 3.3.1 Efficacy evaluation

##### 3.3.1.1 Total effective rate

**Figure 4:**
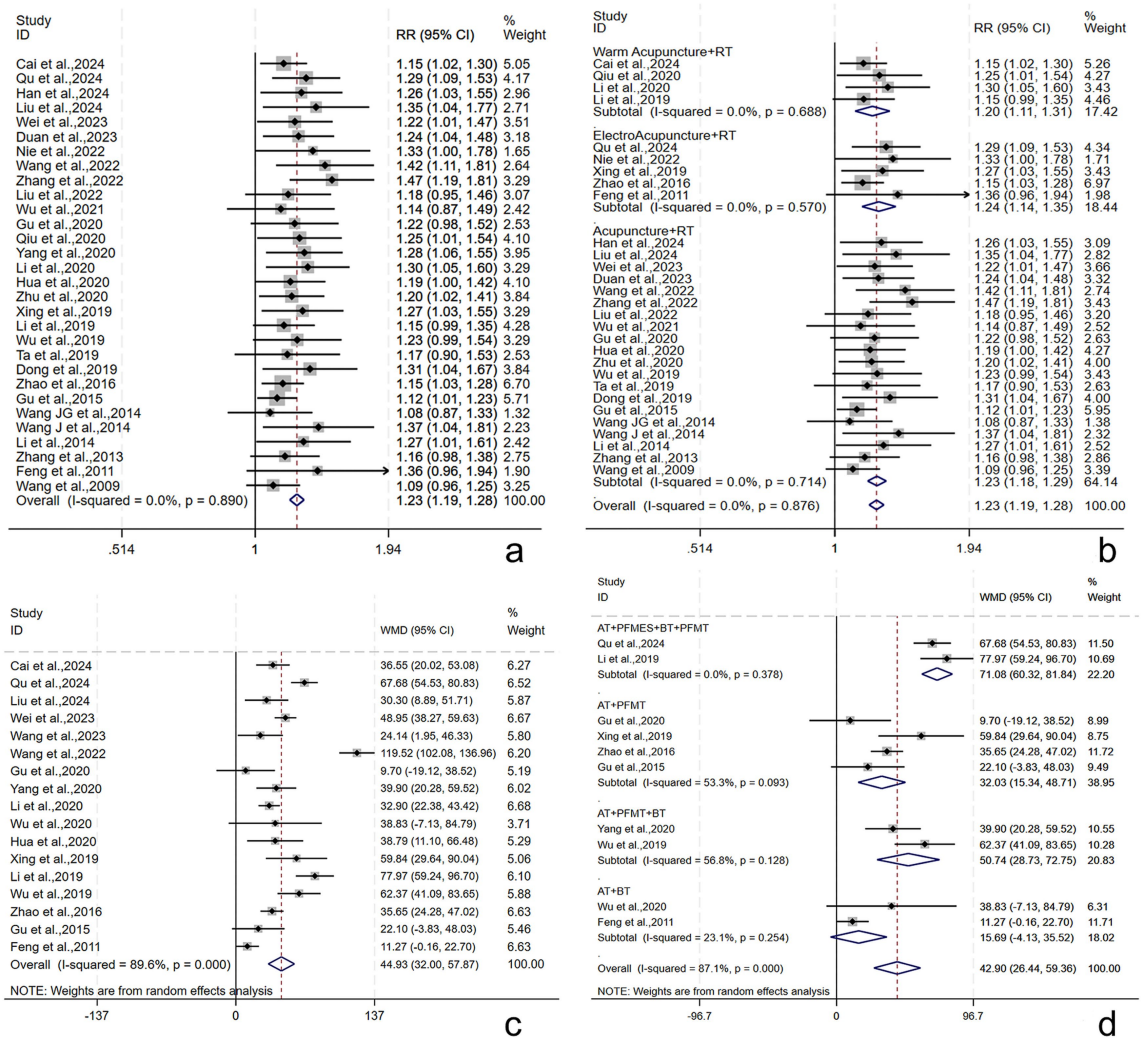
Total effective rate and maximum bladder capacity following AT plus related RT for PSUI. **(a)** Forest plot of total effective rate following AT combined with RT for PSUI. **(b)** Subgroup analysis of total effective rate following AT combined with RT for PSUI, stratified by types of AT. **(c)** Forest plot of maximum bladder capacity following AT combined with RT for PSUI. **(d)** Subgroup analysis of maximum bladder capacity following AT combined with RT for PSUI, stratified by types of RT.

Thirty studies (21, 28, 29, 31, 32, 34–38, 40, 42–47, 49–62) reported the total effective rate, involving 2,432 subjects (1,216/1216: experimental/control group). The intergroup heterogeneity was small (*I*^2^ = 0.00%, *p* = 0.89). Meta-analyses revealed that the effective rate in the experimental group was higher [RR = 1.23, 95% CI (1.19, 1.28), *p* < 0.001], showing a statistically significant difference (Figure 4a). Subgroup analyses were performed by the type of AT, and the effective rates of WA + RT, EA + RT, and A + RT were [RR = 1.20, 95% CI (1.11, 1.31), *p* < 0.001], [RR = 1.24, 95% CI (1.14, 1.35), *p* < 0.001], and [RR = 1.23, 95% CI (1.18, 1.29), *p* < 0.001], respectively (Figure 4b). Possible publication bias was revealed by Egger’s test (*p* = 0.001) and the funnel plot (Supplementary Figure S1). Furthermore, following adjustment by the trim-and-fill method, the pooled effect size remained statistically significant [RR = 1.17, 95% CI (1.13, 1.20), *p* < 0.001], suggesting no great influence of the publication bias on the study results (Supplementary Figure S2). The robustness of the results was verified by sensitivity analyses (Supplementary Figure S3).

#### 3.3.2 Objective indicators of bladder function

##### 3.3.2.1 Maximum bladder capacity

Seventeen studies (21, 29, 31–33, 37, 43, 45, 46, 48–52, 55, 56, 61) reported the maximum bladder capacity, involving 1,487 subjects (744/743: experimental/control group). A random-effects model was utilized (*I*^2^ = 89.6%, *p* < 0.001). The pooled results revealed that the post-treatment maximum bladder capacity in the experimental group was larger [WMD = 44.93, 95% CI (32.00, 57.87), *p* < 0.001], showing a statistically significant difference (Figure 4c). Subgroup analyses were conducted by the treatment course, patient age, stroke type, RT, and AT. AT + PFMES + BT + PFMT, AT + PFMT, AT + BT + PFMT, and AT + BT improved the maximum bladder capacity by [WMD = 71.08, 95% CI (60.32, 81.84), *p* < 0.001], [WMD = 32.03, 95% CI (15.34, 48.71), *p* < 0.001], [WMD = 50.74, 95% CI (28.73, 72.75), *p* < 0.001], and [WMD = 15.69, 95% CI (−4.13, 35.52), *p* = 0.121], respectively (Figure 4d). The results demonstrated that the above subgroups were not a source of heterogeneity (Supplementary Table S1). No publication bias was revealed by Egger’s test (*p* = 0.699) and the funnel plot (Supplementary Figure S4). The robustness of the results was verified by sensitivity analyses (Supplementary Figure S5).

##### 3.3.2.2 RUV

**Figure 5:**
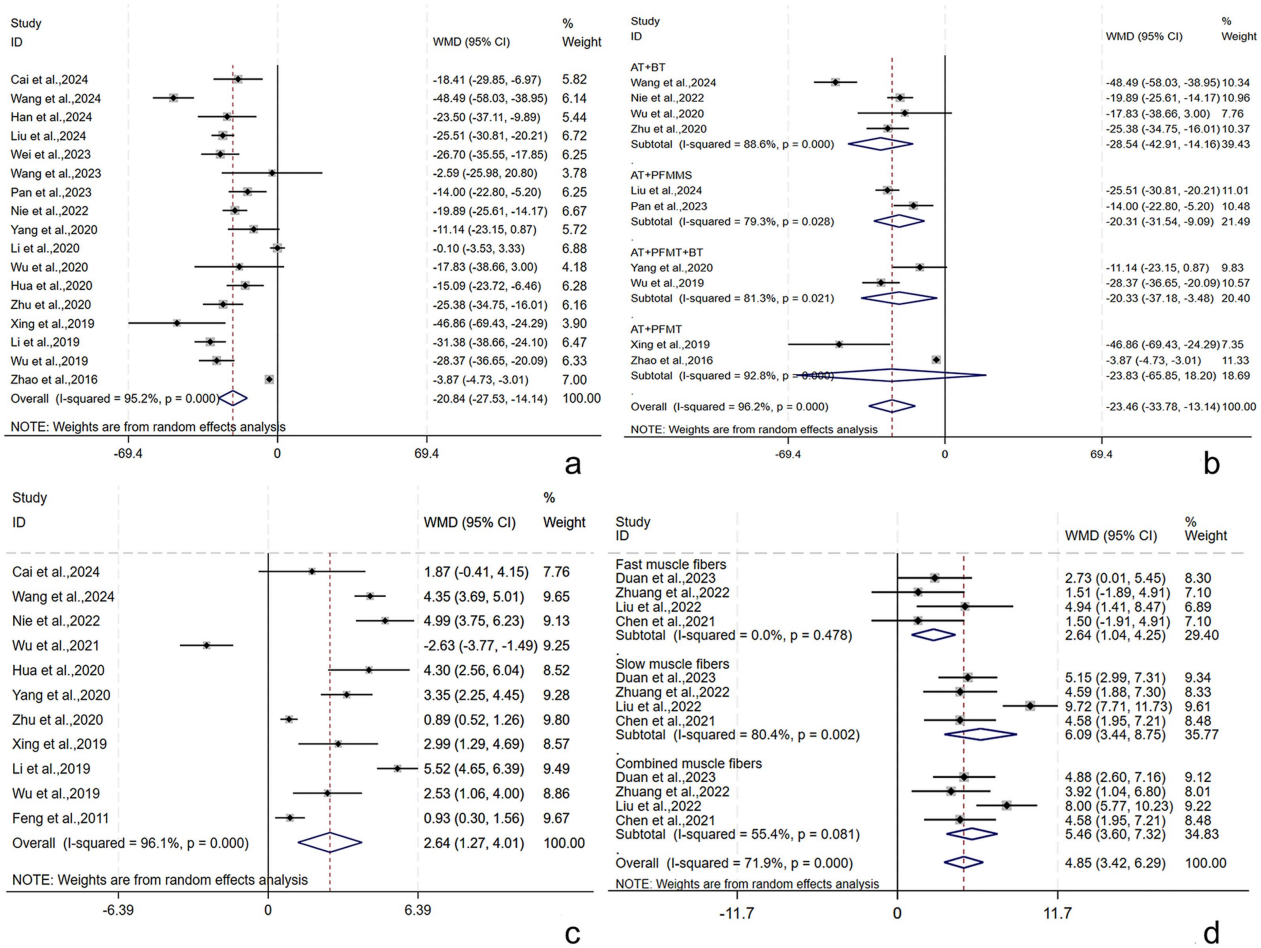
RUV, Qmax, and pelvic floor muscle strength following AT plus related RT for PSUI. **(a)** Forest plot of RUV following AT combined with RT for PSUI. **(b)** Subgroup analysis of RUV following AT combined with RT for PSUI, stratified by types of RT. **(c)** Forest plot of Qmax following AT combined with RT for PSUI. **(d)** Forest plot of pelvic floor muscle strength following AT combined with RT for PSUI.

Seventeen studies (21, 28–30, 32, 33, 35, 36, 45–52, 55) reported the RUV, involving 1,424 subjects (712/712: experimental/control group). A random-effects model was utilized (*I*^2^ = 95.2%, *p* < 0.001). The pooled results revealed that the post-treatment RUV in the experimental group was smaller [WMD = −20.84, 95% CI (−27.53, −14.14), *p* = 0.001], showing a statistically significant difference (Figure 5a). Subgroup analyses were conducted by the treatment course, patient age, stroke type, RT, and AT. AT + BT, AT + PFMMS, AT + BT + PFMT, and AT + PFMT reduced RUV by [WMD = −28.54, 95% CI (−42.91, −14.16), *p* < 0.001], [WMD = −20.31, 95% CI (−31.54, −9.09), *p* < 0.001], [WMD = −20.33, 95% CI (−37.18, −3.48), *p* = 0.018], and [WMD = −23.83, 95% CI (−65.85, 18.20), *p* = 0.266], respectively (Figure 5b). The results demonstrated that the above subgroups were not a source of heterogeneity (Supplementary Table S1). Possible publication bias was revealed by Egger’s test (*p* < 0.001) and the funnel plot (Supplementary Figure S6). Furthermore, following adjustment by the trim-and-fill method, the pooled effect size remained statistically significant, suggesting no great influence of the publication bias on the study results (Supplementary Figure S7). The robustness of the results was verified by sensitivity analyses (Supplementary Figure S8).

##### 3.3.2.3 Qmax

The Qmax was described in 11 studies (29, 30, 36, 42, 45, 47, 49–52, 61) involving 902 subjects (452/450: experimental/control group). A random-effects model was utilized (*I*^2^ = 96.1%, *p* < 0.001). The pooled results showed that the post-treatment Qmax was higher in the experimental group [WMD = 2.64, 95% CI (1.27, 4.01), *p* < 0.001], and the difference was statistically significant (Figure 5c). Subgroup analyses were conducted by the treatment course, patient age, stroke type, RT, and AT. The results demonstrated that the above subgroups were not a source of heterogeneity (Supplementary Table S1). Publication bias was assessed by Egger’s tests and funnel plots (Supplementary Figure S9), and no publication bias was found (*p* = 0.294). The robustness of the findings was verified by the sensitivity analysis (Supplementary Figure S10).

##### 3.3.2.4 Pelvic floor muscle strength

Pelvic floor muscle strength (including fast, slow, and complex muscles) was described in four studies (34, 39–41) involving 249 subjects (124/125: experimental/control group). In terms of the fast muscle strength, the intergroup heterogeneity was small (*I*^2^ = 0.0%, *p* = 0.478), and meta-analyses showed that the experimental group had higher fast muscle strength post-treatment [WMD = 2.64, 95% CI (1.04, 4.25), *p* = 0.001], displaying a statistically significant difference. In terms of the slow and complex muscle strength, the intergroup heterogeneity was substantial (*I*^2^ = 80.4%, *p* = 0.002) (*I*^2^ = 55.4%, *p* = 0.081), and meta-analyses showed that the experimental group had higher slow and complex muscle strength post-treatment [WMD = 6.09, 95% CI (3.44, 8.75), *p* < 0.001] and [WMD = 5.46, 95% CI (3.60, 7.32), *p* < 0.001], displaying statistically significant differences (Figure 5d).

##### 3.3.2.5 MDP

**Figure 6:**
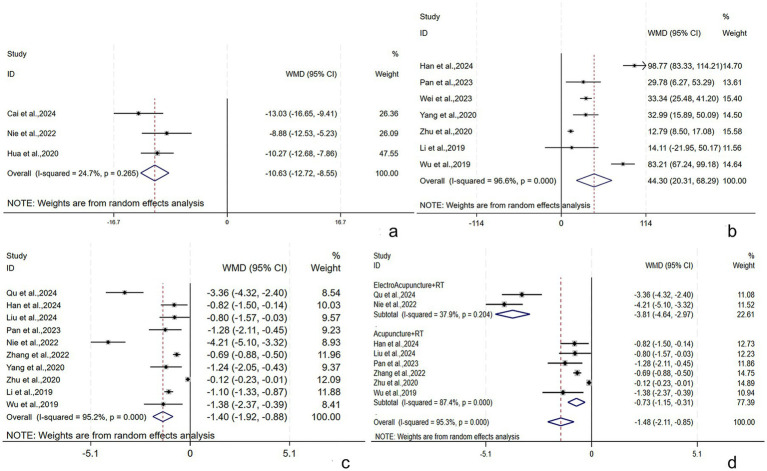
MDP, mean urine output per time, and frequency of 24-h UI following AT plus related RT for PSUI. **(a)** Forest plot of MDP following AT combined with RT for PSUI. **(b)** Forest plot of mean urine output per time following AT combined with RT for PSUI. **(c)** Forest plot of frequency of 24-h UI following AT combined with RT for PSUI. **(d)** Subgroup analysis of frequency of 24-h UI following AT combined with RT for PSUI, stratified by types of AT.

The MDP was described in three studies (29, 36, 49) involving 247 subjects (124/123: experimental/control group). A fixed-effects model was adopted (*I*^2^ = 24.7%, *p* = 0.265). The pooled results showed that the experimental group had lower MDP post-treatment [WMD = −10.6, 95% CI (−12.35, −8.84), *p* = 0.001], and the difference was statistically significant (Figure 6a).

##### 3.3.2.6 Mean urine output per time

Seven studies (28, 32, 34, 45, 47, 51, 52) reported mean urine output per time among 594 subjects (292/292: experimental/control group). A random-effects model was adopted (*I*^2^ = 96.6%, *p* < 0.001). The pooled results found a higher mean urine output per time post-treatment in the experimental group [WMD = 44.30, 95% CI (20.31, 68.29), *p* < 0.001], with a statistically significant difference (Figure 6b). Subgroup analyses were conducted by the treatment course, patient age, and RT. The results demonstrated that the above subgroups were not a source of heterogeneity (Supplementary Table S1).

##### 3.3.2.7 Frequency of 24-h UI

Ten studies (21, 28, 31, 34–36, 38, 45, 47, 51, 52) reported the frequency of 24-h UI among 814 subjects (407/407: experimental/control group). A random-effects model was adopted (*I*^2^ = 95.2%, *p* < 0.001). The pooled results revealed a lower frequency of 24-h UI post-treatment in the experimental group [WMD = −1.40, 95% CI (−1.92, −0.88), *p* < 0.001], showing a statistically significant difference (Figure 6c). Subgroup analyses were conducted by the treatment course, patient age, RT, and AT. EA + RT and A + RT reduced the frequency of 24-h UI by [WMD = −3.81, 95% CI (−4.64, −2.97), *p* = 0.001] and [WMD = −0.73, 95% CI (−1.15, −0.31), *p* < 0.001], respectively (Figure 6d). The results demonstrated that the above subgroups were not a source of heterogeneity (Supplementary Table S1). The robustness of the findings was verified by the sensitivity analysis (Supplementary Figure S11).

##### 3.3.2.8 Frequency of 24-h urination

**Figure 7:**
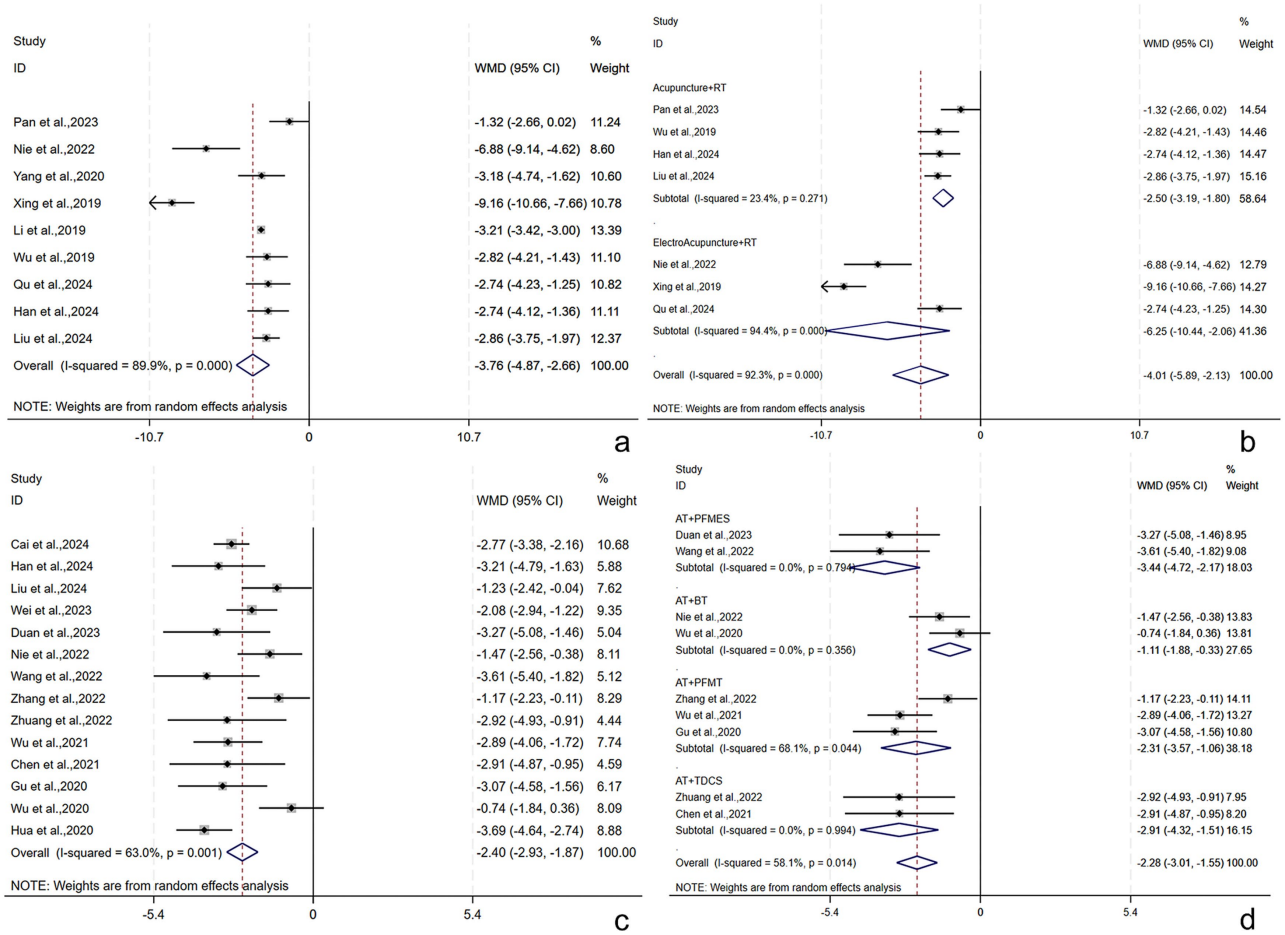
Frequency of 24-h urination and ICIQ-SF scores following AT plus related RT for PSUI. **(a)** Forest plot of frequency of 24-h urination following AT combined with RT for PSUI. **(b)** Subgroup analysis of frequency of 24-h urination following AT combined with RT for PSUI, stratified by types of AT. **(c)** Forest plot of ICIQ-SF scores following AT combined with RT for PSUI. **(d)** Subgroup analysis of ICIQ-SF scores following AT combined with RT for PSUI, stratified by types of RT.

Nine studies (21, 28, 31, 35, 36, 45, 50–52) reported the frequency of 24-h urination among 718 subjects (359/359: experimental/control group). A random-effects model was adopted (*I*^2^ = 89.9%, *p* < 0.001). The pooled results found a lower frequency of 24-h urination post-treatment in the experimental group [WMD = −3.76, 95% CI (−4.87, −2.66), *p* < 0.001], with a statistically significant difference (Figure 7a). Subgroup analyses were conducted by the treatment course, patient age, RT, and AT. EA + RT and A + RT reduced the frequency of 24-h urination by [WMD = −6.25, 95% CI (−10.44, −2.06), *p* = 0.003] and [WMD = -2.50, 95% CI (−3.19, −1.80), *p* < 0.001], respectively (Figure 7b). The results demonstrated that the above subgroups were not a source of heterogeneity (Supplementary Table S1). The robustness of the findings was verified by the sensitivity analysis (Supplementary Figure S12).

#### 3.3.3 Scores of subjective scales of stroke severity, urinary incontinence symptoms, and QOL

##### 3.3.3.1 ICIQ-SF score

The ICIQ-SF score was described in 14 studies (21, 28, 29, 32, 34, 36–39, 41–43, 48, 49) involving 985 subjects (492/493: experimental/control group). A random-effects model was adopted (*I*^2^ = 63.0%, *p* = 0.001). The pooled results revealed that the experimental group had lower ICIQ-SF scores post-treatment [WMD = −2.40, 95% CI (−2.93, −1.83), *p* < 0.001], and the difference was statistically significant (Figure 7c). Subgroup analyses were conducted by the treatment course, patient age, stroke type, RT, and AT. AT + PFMES, AT + BT, AT + PFMT, and AT + TDCS reduced the ICIQ-SF score by [WMD = −3.44, 95% CI (−4.72, −2.17), *p* < 0.001], [WMD = −1.11, 95% CI (−1.88, −0.33), *p* = 0.005], [WMD = −2.31, 95% CI (−3.57, −1.06), *p* < 0.001], and [WMD = −2.91, 95% CI (−4.32, −1.51), *p* < 0.001], respectively (Figure 7d). The results demonstrated that the above subgroups were not a source of heterogeneity (Supplementary Table S1). No publication bias was revealed by Egger’s test (*p* = 0.765) and the funnel plot (Supplementary Figure S13). Sensitivity analyses verified the robustness of the results (Supplementary Figure S14).

##### 3.3.3.2 I-QOL score

**Figure 8:**
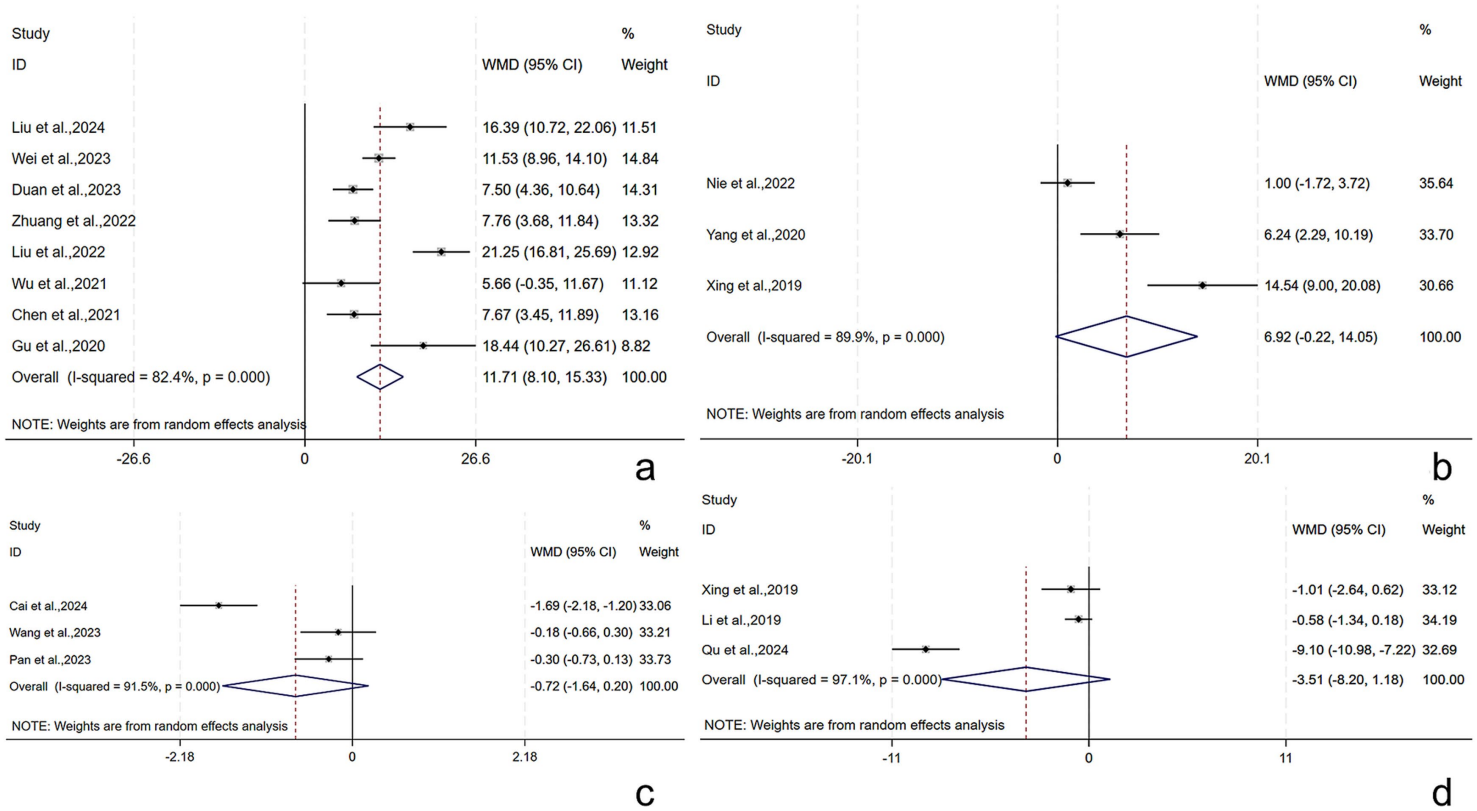
I-QOL, BI, QOL, and NIHSS scores following AT plus related RT for PSUI. **(a)** Forest plot of I-QOL scores following AT combined with RT for PSUI. **(b)** Forest plot of BI scores following AT combined with RT for PSUI. **(c)** Forest plot of QOL scores following AT combined with RT for PSUI. **(d)** Forest plot of NIHSS scores following AT combined with RT for PSUI.

The I-QOL score was reported in eight studies (21, 32, 34, 39–43) involving 533 subjects (266/267: experimental/control group). A random-effects model was utilized (*I*^2^ = 82.4%, *p* < 0.001). The pooled results revealed that the experimental group had higher I-QOL scores post-treatment [WMD = 11.71, 95% CI (8.10, 15.33), *p* < 0.001], with a statistically significant difference (Figure 8a). Subgroup analyses were conducted by the treatment course, patient age, and RT. The results demonstrated that the above subgroups were not a source of heterogeneity (Supplementary Table S1).

##### 3.3.3.3 BI score

The BI score was described in three studies (36, 45, 50) involving 224 subjects (112/112: experimental/control group). A random-effects model was adopted (*I*^2^ = 89.9%, *p* < 0.001). The pooled results revealed that the experimental group had higher BI scores post-treatment [WMD = 6.92, 95% CI (−0.22, 14.05), *p* = 0.058], but the difference was not statistically significant (Figure 8b).

##### 3.3.3.4 QOL score

The QOL score was described in three studies (29, 33, 35) involving 240 subjects (120/120: experimental/control group). A random-effects model was adopted (*I*^2^ = 91.5%, *p* < 0.001). The pooled results revealed that the experimental group had lower QOL scores post-treatment [WMD = −0.72, 95% CI (−1.64, 0.20), *p* = 0.127], without statistically significant difference (Figure 8c).

##### 3.3.3.5 NIHSS score

The NIHSS score was reported in three studies (31, 50, 51) involving 280 patients (140/140: experimental/control group). A random-effects model was adopted (*I*^2^ = 97.1%, *p* < 0.001). The pooled results revealed that the experimental group had lower NIHSS scores post-treatment [WMD = −3.51, 95% CI (−8.20, 1.18), *p* = 0.143], without statistically significant difference (Figure 8d).

## 4 Discussion

This study is the first to meta-analyze the efficacy of AT plus RT on PSUI. A total of 36 studies involving 2,796 PSUI patients were included. The results demonstrated that AT plus related RT improved efficacy, bladder function, related symptoms, and QOL in PSUI patients. However, no significant improvement was observed in the BI, NIHSS score, and QOL scores with AT plus related RT as compared to the control group.

Stroke can cause damage to the central nervous system (64, 65), leading to detrusor overactivity and loss of bladder control (66), ultimately resulting in PSUI. Research suggests that acupuncture at the four sacral points—two upper points located on both sides of the sacrococcygeal joint, approximately 1 cm from the joint, and two lower points located on both sides of the tip of the coccyx, approximately 1 cm from the coccyx—can inhibit central overactivity, thereby alleviating the symptoms of PSUI (67). In addition, acupuncture at local indications in the lower abdomen produces signals that can enter the same or adjacent sacral spinal cord segments, potentially influencing the micturition center of the sacral cord to varying degrees and regulating urinary function. The possible mechanism is that it stimulates the pelvic nerves, hypogastric nerves, and pudendal nerves, which are associated with urination activities (68–70). Acupuncture at relevant points on the head improves the regulation of urination by the cortical center (paracentral lobule) and suppresses excessive reflexive bladder contractions. When the urine volume in the bladder increases, the cortex can send impulses that travel through descending fibers to the low-level spinal centers, inhibiting the parasympathetic center of the sacral cord, stimulating the motor neurons in the anterior horn of the sacral cord and sympathetic centers in the lumbar cord, thereby achieving detrusor relaxation (71). The positive role of RT in PSUI treatment has also been verified. For example, PFMT can increase pelvic floor muscle strength and enhance momentary control of urethral closure pressure in patients with UI, helping them gradually build up the ability of autonomous conscious control of pelvic floor muscle groups (72), thereby significantly reducing the frequency of daytime urination and UI (73). PFMES coordinates bladder muscle contraction and urethral sphincter relaxation by stimulating the corresponding innervating nerve of pelvic muscles, thereby increasing bladder capacity (74) and improving bladder compliance (75). A clinical trial found that neuromuscular electrical stimulation plus BT can ameliorate the leakage of urine, urological symptoms, and QOL in women with PSUI (15). As can be inferred from these studies, AT plus RT can contribute positively to the recovery of PSUI patients at both the neurological and local tissue levels (39, 48, 49); that is, it can enhance neurological remodeling to improve the control of urination by higher centers, increase the bladder capacity to reduce the frequency of urination, promote the blood circulation and viability of pelvic floor muscles, and boost the oxygen supply and metabolism of local muscle tissues, thereby relieving UI (43, 50).

We conducted a subgroup analysis based on the type of AT and found that WA + RT, EA + RT, and A + RT had higher total effective rates than the control group; however, WA + RT was inferior to the others. After excluding the methodological bias and reviewing the relevant articles (76, 77), we concluded that this trend was possibly related to the insufficient intensity of WA (controlled amount and duration of warm stimulation in WA).

The subgroup analysis of the maximum bladder capacity showed that the increase in the maximum bladder capacity was correlated with the number of RTs in the intervention and that AT + PFMES + BT + PFMT achieved the greatest recovery of the maximum bladder capacity. We found that when only one RT was included, AT + PFMT outperformed AT + BT in improving maximum bladder capacity. The reason is that PFMES is usually given under the guidance of rehabilitation professionals, making it a more standardized and scientific process, thereby increasing the stability and reproducibility of its efficacy. In contrast, BT partly relies on the patient’s subjective adherence, which may lead to bias in the training effect due to individual differences and insufficient compliance.

AT + PFMT did not significantly improve RUV, whereas AT + BT yielded the most significant improvement in RUV. This finding is similar to that of a clinical trial on overactive bladder syndrome, which reported no significant difference between BT and BT + PFMT in improving the outcome metrics for bladder function (78).

The subgroup analysis by the type of AT revealed that EA + RT was superior to A + RT in reducing the frequency of 24-h UI and the frequency of 24-h urination. After reviewing the included studies and related studies (79–82), we believed that this difference may be related to the stability of the stimulations. The efficacy of A depends largely on the operation skills of the acupuncturist, so it may be affected by personal factors such as the acupuncturist’s experience and proficiency in operation, resulting in certain variability in efficacy. In contrast, the intensity of EA is controlled by the EA apparatus, ensuring a stable and continuous stimulation effect. This helps reduce bias caused by differences in the acupuncturist’s operation. With a standardized mode of stimulation, EA may achieve more consistent efficacy in some studies.

For the ICIQ-SF score, the subgroup analyses demonstrated that AT + PFMES was superior to AT + TDCS, AT + PFMT, and AT + BT. A meta-analysis on overactive bladder syndrome (83) showed no significant difference between PFMT and BT display in improving the QOL, which is consistent with our findings. The reason is that this study only focused on the ICIQ-SF score and adopted not exactly the same interventions. The effect of AT + PFMES on ICIQ-SF scores drew our attention. As a non-invasive treatment, PFMES is characterized by significant efficacy and adjustable parameters (84, 85), making it highly attractive in clinical application. In the future, in-depth research is further required to clarify the true clinical value of PFMES.

In addition, AT plus related RT made no great improvement in the QOL, NIHSS, and BI scores compared with controls. QOL, NIHSS, and BI scores (used for assessing patients’ activities of daily living and neurological deficits in stroke) are influenced by stroke-induced motor dysfunction, sensory disorders, emotional changes, and psychosocial changes (86, 87), but related symptoms cannot be fully ameliorated by the intervention, thus no great changes occur in QOL, NIHSS, and BI scores.

We performed subgroup analyses to explore the possible source of great heterogeneity in the maximum bladder capacity, RUV, Qmax, mean urine output per time, frequency of 24-h UI, frequency of 24-h urination, ICIQ-SF score, and I-QOL score. However, no source of heterogeneity was found. We believe that the considerable heterogeneity may result from differences in intervention details—such as the difficulty in completely standardizing the microoperation, including the intensity and operation techniques of AT—variability in individual responses (some of the RCTs included did not report TCM syndrome differentiation typing, so physical differences may have amplified the dispersion of efficacy, hindering further exploration).

Despite a detailed meta-analysis of AT plus RT for treating PSUI, some limitations are still present. In particular, the overall methodological quality of the included studies was low, which may have led to larger errors. Future studies should focus on improving the evidence-based quality to minimize bias and enhance the reliability of results. Additionally, only one eligible English-language study was retrieved from the eight databases, and all populations were from China, which could produce heterogeneity in the study population. As a result, the generalization of the findings is limited.

## 5 Conclusion

AT plus related RT can ameliorate clinical efficacy, bladder function, related symptoms, and QOL in PSUI patients. However, some limitations are still present in this study. Further high-quality clinical studies are required to validate these findings in the future, with particular attention to evaluating the efficacy across diverse ethnic groups, thereby providing more solid clinical evidence.

## Data Availability

The original contributions presented in the study are included in the article/Supplementary material, further inquiries can be directed to the corresponding author.
