# Supplementary material for: Efficacy of acupuncture therapy plus related rehabilitation therapy for post-stroke urinary incontinence: a systematic review and meta-analysis
Source: Front Neurol. 2025 May 8;16:1575970. doi: 10.3389/fneur.2025.1575970 (PMC12108803; doi:10.3389/fneur.2025.1575970)
Supplement: Supplementary file 1 [file Supplementary_file_1.docx]

**Search strategy table**

| **Pubmed** | |
| --- | --- |
| # | Query |
| 1 | "Stroke"[Mesh] |
| 2 | "acute focal cerebral vasculopathy” OR “acutestroke*” OR “apolex*” OR “apoplex*” OR “brain accident*” OR “brain attack*” OR “brain blood flow disturbance” OR “brain infarct*” OR “brain insult*” OR “brain vascular accident*” OR “brain venous infarction*” OR “cerebral circulation infarction*” OR “cerebral insult*” OR “cerebral vascular accident*” OR “cerebral vascular insufficienc*” OR “cerebralstroke*” OR “cerebro vascular accident*” OR “cerebrovascular accident*” OR “cerebrovascular arrest*” OR “cerebrovascular failure*” OR “cerebrovascular injur*” OR “cerebrovascular insufficienc*” OR “cerebrovascular insult*” OR “cerebrovascular lesion*” OR “cerebrum vascular accident*” OR “circulation brain infarction*” OR “cva*” OR “stroke*” OR “venous brain infarction*" |
| 3 | #1 OR #2 |
| 4 | "Urinary Incontinence"[Mesh] |
| 5 | "bladder incontinence” OR “incontinentia urinae” OR “involuntary urinary loss” OR “involuntary urination” OR “involuntary urine loss” OR “leakage of urine” OR “unwanted urine loss” OR “urinary incontinence” OR “urinary leakage” OR “urinary reflex incontinence” OR “urinary stress incontinence” OR “urinary urge incontinence” OR “urine bladder incontinence” OR “urine incontinence” OR “urine leakage" |
| 6 | #4 OR #5 |
| 7 | "Acupuncture Therapy"[Mesh] |
| 8 | "acupoint*” OR “acupuncture” OR “electroacupuncture” OR “jing luo” OR “jing mai” OR “jingluo” OR “jingmai” OR “meridian*” OR “pharmacoacupuncture” OR “shonishin” OR “earacupouncture" |
| 9 | #7 OR #8 |
| 10 | "Randomized Controlled Trials as Topic"[Mesh] |
| 11 | "random*" |
| 12 | #10 OR #11 |
| 13 | #3 AND #6 AND #9 AND #12 |

| **Embase** | |
| --- | --- |
| # | Query |
| 1 | 'cerebrovascular accident'/exp |
| 2 | 'acute focal cerebral vasculopathy’ OR ‘acutestroke*’ OR ‘apolex*’ OR ‘apoplex*’ OR ‘brain accident*’ OR ‘brain attack*’ OR ‘brain blood flow disturbance’ OR ‘brain infarct*’ OR ‘brain insult*’ OR ‘brain vascular accident*’ OR ‘brain venous infarction*’ OR ‘cerebral circulation infarction*’ OR ‘cerebral insult*’ OR ‘cerebral vascular accident*’ OR ‘cerebral vascular insufficienc*’ OR ‘cerebralstroke*’ OR ‘cerebro vascular accident*’ OR ‘cerebrovascular accident*’ OR ‘cerebrovascular arrest*’ OR ‘cerebrovascular failure*’ OR ‘cerebrovascular injur*’ OR ‘cerebrovascular insufficienc*’ OR ‘cerebrovascular insult*’ OR ‘cerebrovascular lesion*’ OR ‘cerebrum vascular accident*’ OR ‘circulation brain infarction*’ OR ‘cva*’ OR ‘stroke*’ OR ‘venous brain infarction*' |
| 3 | #1 OR #2 |
| 4 | 'urine incontinence'/exp |
| 5 | 'bladder incontinence’ OR ‘incontinentia urinae’ OR ‘involuntary urinary loss’ OR ‘involuntary urination’ OR ‘involuntary urine loss’ OR ‘leakage of urine’ OR ‘unwanted urine loss’ OR ‘urinary incontinence’ OR ‘urinary leakage’ OR ‘urinary reflex incontinence’ OR ‘urinary stress incontinence’ OR ‘urinary urge incontinence’ OR ‘urine bladder incontinence’ OR ‘urine incontinence’ OR ‘urine leakage' |
| 6 | #4 OR #5 |
| 7 | 'acupuncture'/exp |
| 8 | acupoint*’ OR ‘acupuncture’ OR ‘electroacupuncture’ OR ‘jing luo’ OR ‘jing mai’ OR ‘jingluo’ OR ‘jingmai’ OR ‘meridian*’ OR ‘pharmacoacupuncture’ OR ‘shonishin’ OR ‘earacupouncture' |
| 9 | #7 OR #8 |
| 10 | 'randomized controlled trial'/exp |
| 11 | 'random*' |
| 12 | #10 OR #11 |
| 13 | #3 AND #6 AND #9 AND #12 |
| **Cochrane** | |
| # | Query |
| 1 | MeSH descriptor: [Stroke] explode all trees |
| 2 | ('acute focal cerebral vasculopathy' OR 'acutestroke*' OR 'apolex*' OR 'apoplex*' OR 'brain accident*' OR 'brain attack*' OR 'brain blood flow disturbance' OR 'brain infarct*' OR 'brain insult*' OR 'brain vascular accident*' OR 'brain venous infarction*' OR 'cerebral circulation infarction*' OR 'cerebral insult*' OR 'cerebral vascular accident*' OR 'cerebral vascular insufficienc*' OR 'cerebralstroke*' OR 'cerebro vascular accident*' OR 'cerebrovascular accident*' OR 'cerebrovascular arrest*' OR 'cerebrovascular failure*' OR 'cerebrovascular injur*' OR 'cerebrovascular insufficienc*' OR 'cerebrovascular insult*' OR 'cerebrovascular lesion*' OR 'cerebrum vascular accident*' OR 'circulation brain infarction*' OR 'cva*' OR 'stroke*' OR 'venous brain infarction*') |
| 3 | #1 OR #2 |
| 4 | MeSH descriptor: [Urinary Incontinence] explode all trees |
| 5 | ('bladder incontinence' OR 'incontinentia urinae' OR 'involuntary urinary loss' OR 'involuntary urination' OR 'involuntary urine loss' OR 'leakage of urine' OR 'unwanted urine loss' OR 'urinary incontinence' OR 'urinary leakage' OR 'urinary reflex incontinence' OR 'urinary stress incontinence' OR 'urinary urge incontinence' OR 'urine bladder incontinence' OR 'urine incontinence' OR 'urine leakage') |
| 6 | #4 OR #5 |
| 7 | MeSH descriptor: [Acupuncture] explode all trees |
| 8 | ('acupoint*' OR 'acupuncture' OR 'electroacupuncture' OR 'jing luo' OR 'jing mai' OR 'jingluo' OR 'jingmai' OR 'meridian*' OR 'pharmacoacupuncture' OR 'shonishin' OR 'earacupouncture') |
| 9 | #7 OR #8 |
| 10 | MeSH descriptor: [Randomized Controlled Trial] explode all trees |
| 11 | ('random*') |
| 12 | #10 OR #11 |
| 13 | #3 AND #6 AND #9 AND #12 |

| **Web of Science** | |
| --- | --- |
| # | Query |
| 1 | TS=((acute focal cerebral vasculopathy) OR (acutestroke*) OR (apolex*) OR (apoplex*) OR (brain accident*) OR (brain attack*) OR (brain blood flow disturbance) OR (brain infarct*) OR (brain insult*) OR (brain vascular accident*) OR (brain venous infarction*) OR (cerebral circulation infarction*) OR (cerebral insult*) OR (cerebral vascular accident*) OR (cerebral vascular insufficienc*) OR (cerebralstroke*) OR (cerebro vascular accident*) OR (cerebrovascular accident*) OR (cerebrovascular arrest*) OR (cerebrovascular failure*) OR (cerebrovascular injur*) OR (cerebrovascular insufficienc*) OR (cerebrovascular insult*) OR (cerebrovascular lesion*) OR (cerebrum vascular accident*) OR (circulation brain infarction*) OR (cva*) OR (stroke*) OR (venous brain infarction*)) |
| 2 | TS=((bladder incontinence) OR (incontinentia urinae) OR (involuntary urinary loss) OR (involuntary urination) OR (involuntary urine loss) OR (leakage of urine) OR (unwanted urine loss) OR (urinary incontinence) OR (urinary leakage) OR (urinary reflex incontinence) OR (urinary stress incontinence) OR (urinary urge incontinence) OR (urine bladder incontinence) OR (urine incontinence) OR (urine leakage)) |
| 3 | TS=((acupoint*) OR (acupuncture) OR (electroacupuncture) OR (jing luo) OR (jing mai) OR (jingluo) OR (jingmai) OR (meridian*) OR (pharmacoacupuncture) OR (shonishin) OR (earacupouncture)) |
| 4 | TS=((randomized controlled trial*) OR (random*)) |
| 5 | #1 AND #2 AND #3 AND #4 |

| **China National Knowledge Infrastructure (CNKI)** | |
| --- | --- |
| # | Query |
| 1 | (SU=卒中 OR SU=脑卒中 OR SU=中风 OR SU=脑出血 OR SU=脑缺血 OR SU=脑出血 OR SU=缺血性脑卒中 OR SU=出血性脑卒中 OR SU=脑梗死) |
| 2 | (SU=尿失禁 OR SU=漏尿 OR SU=盆底肌 OR SU=盆底障碍性疾病 OR SU=小便失禁 OR SU=失禁 OR SU=盆底功能障碍) |
| 3 | (SU=针刺 OR SU=针灸 OR SU=毫针 OR SU=电针 OR SU=耳针 OR SU=头针 OR SU=腹针 OR SU=火针 OR SU=穴位 OR SU=针法) |
| 4 | (SU=随机) |
| 5 | #1 AND #2 AND #3 AND #4 |

| **Wanfang Digital Periodicals database** | |
| --- | --- |
| # | Query |
| 1 | (主题=卒中 OR 主题=脑卒中 OR 主题=中风 OR 主题=脑出血 OR 主题=脑缺血OR 主题=缺血性脑卒中 OR 主题=出血性脑卒中 OR 主题=脑梗死) |
| 2 | (主题=尿失禁 OR 主题=漏尿 OR 主题=盆底肌 OR 主题=盆底障碍性疾病 OR 主题=小便失禁 OR 主题=失禁) |
| 3 | (主题=针刺 OR 主题=针灸 OR 主题=毫针 OR 主题=电针 OR 主题=耳针 OR 主题=头针 OR 主题=腹针 OR 主题=火针 OR 主题=穴位 OR 主题=针法) |
| 4 | (主题=随机) |
| 5 | #1 AND #2 AND #3 AND #4 |

| **Chinese Science and Technology Periodicals database** | |
| --- | --- |
| # | Query |
| 1 | U=(针刺+针灸+毫针+电针+耳针+头针+腹针+火针+穴位+针法) |
| 2 | U=(卒中+脑卒中+中风+脑出血+脑缺血+缺血性脑卒中+出血性脑卒中+脑梗死) |
| 3 | U=(尿失禁+漏尿+盆底肌+盆底障碍性疾病+小便失禁+失禁) |
| 4 | U=(随机) |
| 5 | #1 AND #2 AND #3 AND #4 |

| **Chinese biomedical literature service system** | |
| --- | --- |
| # | Query |
| 1 | “针刺”[常用字段:智能] OR “针灸”[常用字段:智能] OR “毫针”[常用字段:智能] OR “电针”[常用字段:智能] OR “耳针”[常用字段:智能] OR “头针”[常用字段:智能] OR “腹针”[常用字段:智能] OR “火针”[常用字段:智能] OR “穴位”[常用字段:智能] OR “针法”[常用字段:智能] |
| 2 | “卒中”[常用字段:智能] OR “脑卒中”[常用字段:智能] OR “中风”[常用字段:智能] OR “脑出血”[常用字段:智能] OR “脑缺血”[常用字段:智能] OR “缺血性脑卒中”[常用字段:智能] OR “出血性脑卒中”[常用字段:智能] OR “脑梗死”[常用字段:智能] |
| 3 | “尿失禁”[常用字段:智能] OR “漏尿”[常用字段:智能] OR “盆底肌”[常用字段:智能] OR “盆底障碍性疾病”[常用字段:智能] OR “小便失禁”[常用字段:智能] OR “失禁”[常用字段:智能] |
| 4 | “随机”[常用字段:智能] |
| 5 | #1 AND #2 AND #3 AND #4 |

**Table S1. Subgroup analysis of acupuncture combined with related rehabilitation therapy for PSUI**

| Outcomes | Group | | Number of trails | Effect Size | | Heterogeneity | |
| --- | --- | --- | --- | --- | --- | --- | --- |
|  |  |  |  | WMD | 95%CI | I² (%) | *P* |
| Maximum bladder capacity | Course of treatment | ＞4wk | 5 | 44.88 | 29.96 , 59.80 | 79 | 0.001 |
|  |  | ＜4wk | 12 | 44.96 | 26.18 , 63.74 | 91.8 | 0.000 |
|  | Age | ＜60year-old | 8 | 52.67 | 29.22 , 76.12 | 91.5 | 0.000 |
|  |  | ＞60year-old | 8 | 42.05 | 30.15 , 53.06 | 46.78 | 0.000 |
|  | Cerebral infarction | Brain Infarction | 5 | 42.8 | 25.05 , 60.54 | 77.9 | 0.001 |
|  |  | Stroke | 12 | 45.85 | 28.60 , 63.11 | 91.9 | 0.000 |
|  | Type of treatment (classified by rehabilitation) | AT+PFMES+BT+PFMT | 2 | 71.08 | 60.32 , 81.84 | 0 | 0.378 |
|  |  | AT+PFMT | 4 | 32.03 | 15.34 , 48.71 | 53.3 | 0.093 |
|  |  | AT+PFMT+BT | 2 | 50.74 | 28.73 , 72.75 | 56.8 | 0.128 |
|  |  | AT+BT | 2 | 15.69 | -4.13 , 35.52 | 23.1 | 0.254 |
|  | Type of treatment (classified by acupuncture therapy) | WA+RT | 3 | 48.32 | 22.66 , 73.98 | 88.5 | 0.000 |
|  |  | EA+RT | 4 | 42.54 | 15.73 , 69.35 | 93.0 | 0.000 |
|  |  | A+RT | 9 | 44.83 | 21.82 , 67.83 | 90.4 | 0.000 |
| Residual urine volume | Course of treatment | ＞4wk | 6 | -20.72 | -32.67 , -8.77 | 96.1 | 0.000 |
|  |  | ＜4wk | 11 | -21.04 | -31.41 , 10.67 | 93.5 | 0.000 |
|  | Age | ＜60year-old | 8 | -22.91 | -28.79 , -17.02 | 61.0 | 0.012 |
|  |  | ＞60year-old | 9 | -19.09 | -2.83 , -10.35 | 96.1 | 0.000 |
|  | Cerebral infarction | Brain Infarction | 4 | -16.96 | -31.66 , -2.25 | 95.4 | 0.000 |
|  |  | Stroke | 12 | -22.47 | -32.24 , -12.60 | 93.4 | 0.000 |
|  | Type of treatment (classified by rehabilitation) | AT +BT | 4 | -28.54 | -42.91 , -14.16 | 88.8 | 0.000 |
|  |  | AT+PFMMS | 2 | -20.31 | -31.54 , -9.09 | 79.3 | 0.028 |
|  |  | AT+PFMT+BT | 2 | -20.33 | -37.18 , -3.48 | 81.3 | 0.021 |
|  |  | AT+PFMT | 2 | -23.83 | -65.85 , -18.20 | 92.8 | 0.000 |
|  | Type of treatment (classified by acupuncture therapy) | WA+RT | 3 | -16.44 | -38.56 , -5.68 | 96.8 | 0.000 |
|  |  | A+RT | 10 | -24.21 | -30.70 , -17.73 | 77.0 | 0.000 |
|  |  | EA+RT | 3 | -19.53 | -35.58 , 3.47 | 95.4 | 0.000 |
| Maximum flow rate | Course of treatment | ＞4wk | 5 | 2.13 | -0.6 , 4.86 | 97.7 | 0.000 |
|  |  | ＜4wk | 6 | 3.04 | 1.60 , 4.49 | 91.5 | 0.000 |
|  | Age | ＜60year-old | 5 | 4.17 | 2.97 , 5.38 | 70.6 | 0.009 |
|  |  | ＞60year-old | 5 | 1.70 | -0.42 , 3.83 | 97.2 | 0.000 |
|  | Cerebral infarction | Brain Infarction | 3 | 4.12 | 2.17 , 6.08 | 78.2 | 0.010 |
|  |  | Stroke | 7 | 1.76 | 0.26 , 3.26 | 96.0 | 0.000 |
|  | Type of treatment (classified by rehabilitation) | AT +BT | 4 | 2.74 | 0.81 , 4.67 | 9.3 | 0.000 |
|  |  | AT+PFMT | 2 | 0.14 | -5.36 , 5.65 | 96.6 | 0.000 |
|  |  | AT+PFMT+BT | 2 | 3.06 | 2.18 , 3.94 | 0 | 0.382 |
|  | Type of treatment (classified by acupuncture therapy) | WA+RT | 2 | 3.85 | 0.29 , 7.42 | 88.4 | 0.003 |
|  |  | A+RT | 5 | 1.86 | -0.39 , 4.11 | 97.2 | 0.000 |
|  |  | EA+RT | 3 | 2.93 | 0.2 , 5.67 | 94.2 | 0.000 |
| Average urine volume | Course of treatment | ＞4wk | 3 | 61.77 | 27.72 , 95.82 | 93.3 | 0.000 |
|  |  | ＜4wk | 4 | 21.42 | -3.78 , 39.05 | 90.1 | 0.000 |
|  | Age | ＜60year-old | 3 | 50.13 | -0.08 , 100.33 | 96.5 | 0.000 |
|  |  | ＞60year-old | 4 | 39.4 | 5.78 , 73.19 | 95.9 | 0.000 |
|  | Type of treatment (classified by rehabilitation) | AT +BT+PFMES+PFMT | 2 | 58.07 | -24.83 , 140.98 | 94.4 | 0.000 |
|  |  | AT+PFMT+BT | 2 | 58.20 | 8.96 , 107.41 | 9.43 | 0.000 |
| Number of incontinence cases within 24h | Course of treatment | ＞4wk | 4 | -1.33 | -2.07 , -0.59 | 97.9 | 0.000 |
|  |  | ＜4wk | 6 | -1.44 | -2.14 , -0.75 | 76.3 | 0.001 |
|  | Age | ＜60year-old | 4 | -1.56 | -2.34 , -0.79 | 95.0 | 0.000 |
|  |  | ＞60year-old | 6 | -1.32 | -2.25 , -0.39 | 92.2 | 0.000 |
|  | Type of treatment (classified by rehabilitation) | AT +BT+PFMT+PFMES | 3 | -1.68 | -2.80 , -0.55 | 90.6 | 0.000 |
|  |  | AT+PFMMS | 2 | -1.02 | -1.59 , -0.45 | 0 | 0.407 |
|  |  | AT+BT | 2 | -2.14 | -6.15 , 1.87 | 98.7 | 0.000 |
|  |  | AT+PFMT+BT | 2 | -1.30 | -1.92 , -0.67 | 0 | 0.830 |
|  | Type of treatment (classified by acupuncture therapy) | EA+RT | 2 | -3.81 | -4.64 , -2.97 | 37.9 | 0.204 |
|  |  | A+RT | 6 | -0.73 | -1.15 , -0.31 | 87.4 | 0.000 |
| Number of urination cases within 24h | Course of treatment | ＞4wk | 2 | -4.86 | -8.44 , -1.29 | 90 | 0.002 |
|  |  | ＜4wk | 7 | -3.53 | -5.23 , -1.82 | 91.4 | 0.000 |
|  | Age | ＜60year-old | 4 | -5.42 | -8.24 , -2.6 | 95.7 | 0.000 |
|  |  | ＞60year-old | 5 | -2.6 | -3.2 , -2 | 11 | 0.343 |
|  | Type of treatment (classified by rehabilitation) | AT +BT+PFMT+PFMES | 3 | -3.19 | -3.40 , -2.98 | 0 | 0.673 |
|  |  | AT+PFMMS | 2 | -2.17 | -3.67 , -0.67 | 71.5 | 0.061 |
|  |  | AT+PFMT+BT | 2 | -2.98 | -4.02 , -1.94 | 0 | 0.736 |
|  | Type of treatment (classified by acupuncture therapy) | A+RT | 4 | -2.50 | -3.19 , -1.8 | 23.4 | 0.271 |
|  |  | EA+RT | 3 | -6.25 | -10.44 , -2.06 | 94.4 | 0.000 |
| ICIQSF score | Course of treatment | ＞4wk | 6 | -2.22 | -2.84 , -1.6 | 55.5 | 0.047 |
|  |  | ＜4wk | 8 | -2.61 | -3.53 , -1.68 | 70.2 | 0.001 |
|  | Age | ＜60year-old | 11 | -2.63 | -3.16 , -2.09 | 51.9 | 0.025 |
|  |  | ＞60year-old | 3 | -1.61 | -2.89 , -0.33 | 73.1 | 0.024 |
|  | Cerebral infarction type | Brain Infarction | 3 | -3.12 | -3.74 , -2.50 | 24 | 0.260 |
|  |  | Stroke | 10 | -2.22 | -2.84 , -1.59 | 55.4 | 0.017 |
|  | 1 | AT +BT | 2 | -1.11 | -1.88 , -0.33 | 0 | 0.356 |
|  |  | AT+PFMES | 2 | -3.44 | -4.72 , -2.17 | 0 | 0.794 |
|  |  | AT+TDCS | 2 | -2.91 | -4.32 , -1.51 | 0 | 0.994 |
|  |  | AT+PFMT | 3 | -2.31 | -3.57 , -1.06 | 68.1 | 0.044 |
| I-QOL score | Course of treatment | ＞4wk | 3 | 8.81 | 5.34 , 12.27 | 63.8 | 0.063 |
|  |  | ＜4wk | 5 | 14.04 | 8.04 , 20.05 | 86.1 | 0.000 |
|  | Age | ＜60year-old | 5 | 9.63 | 6.87 , 12.40 | 59.8 | 0.041 |
|  |  | ＞60year-old | 3 | 14.60 | 5.67 , 23.54 | 88.1 | 0.000 |
|  | Type of treatment (classified by rehabilitation and acupuncture) | A +PFMES | 2 | 14.28 | 0.81 , 27.76 | 95.9 | 0.000 |
|  |  | A+PFMT | 2 | 11.74 | -0.77 , 24.25 | 85.9 | 0.014 |
|  |  | AT+TDCS | 2 | 7.72 | 4.78 , 10.65 | 0 | 0.976 |


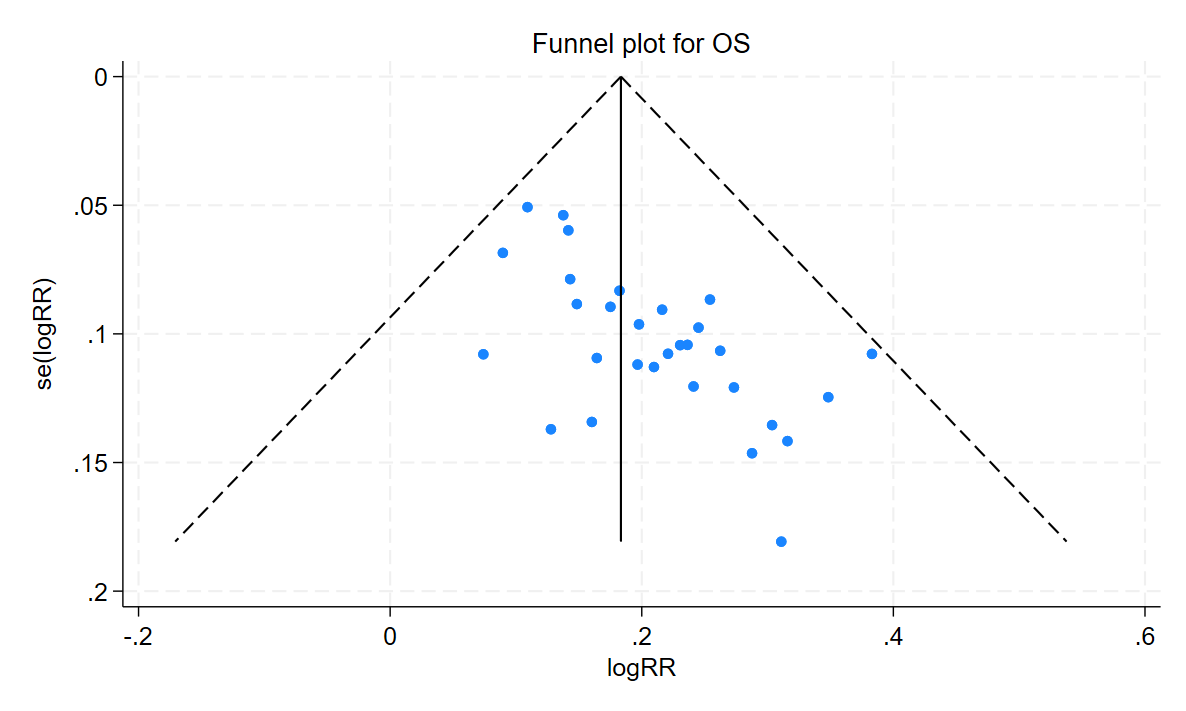


**Figure S1.** Funnel plot of the total effective rate of acupuncture combined with related rehabilitation therapy for PSUI


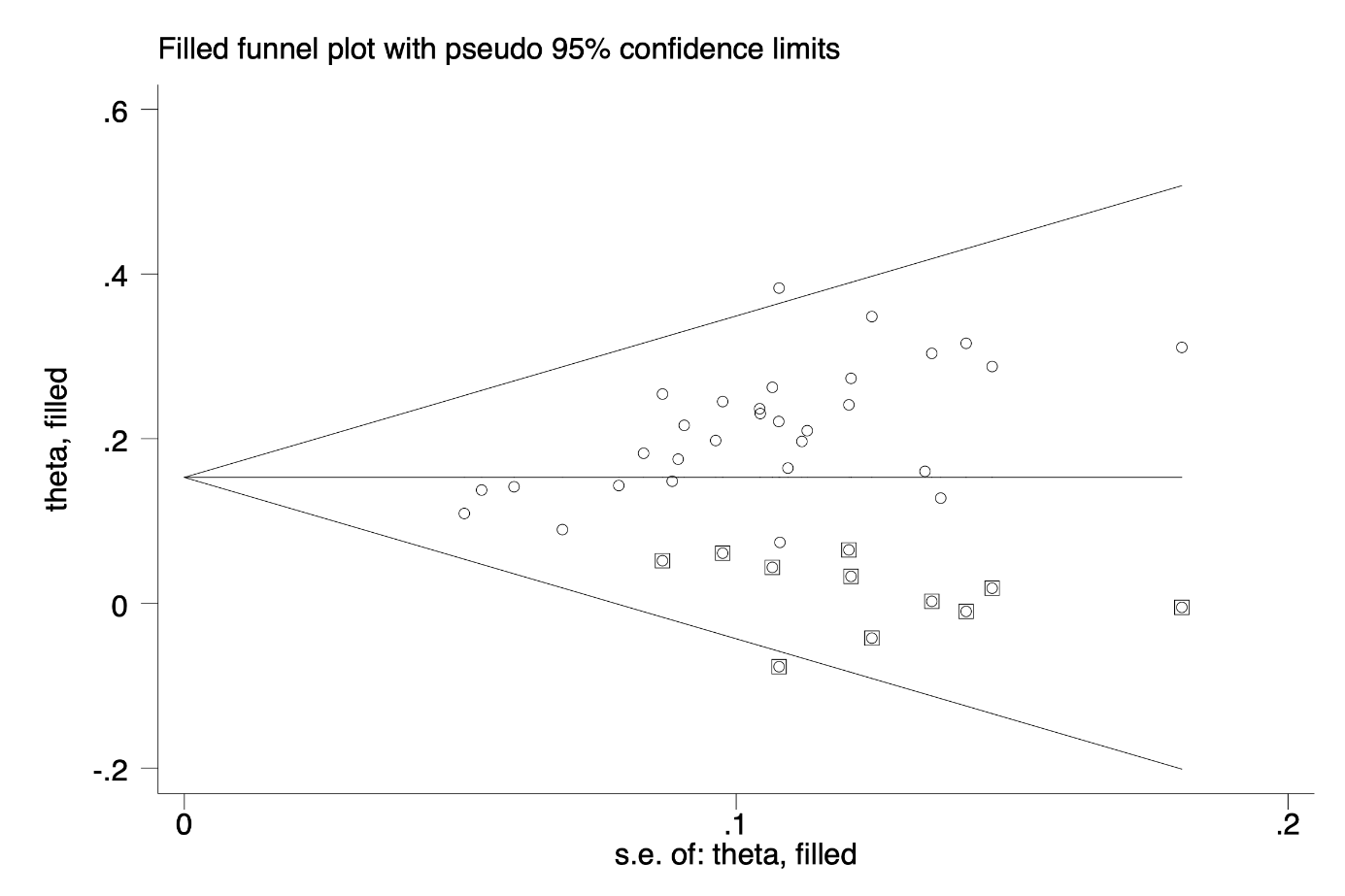


**Figure S2.** The results of the trim and fill method for total effective rate of acupuncture combined with related rehabilitation therapy in the treatment of PSUI


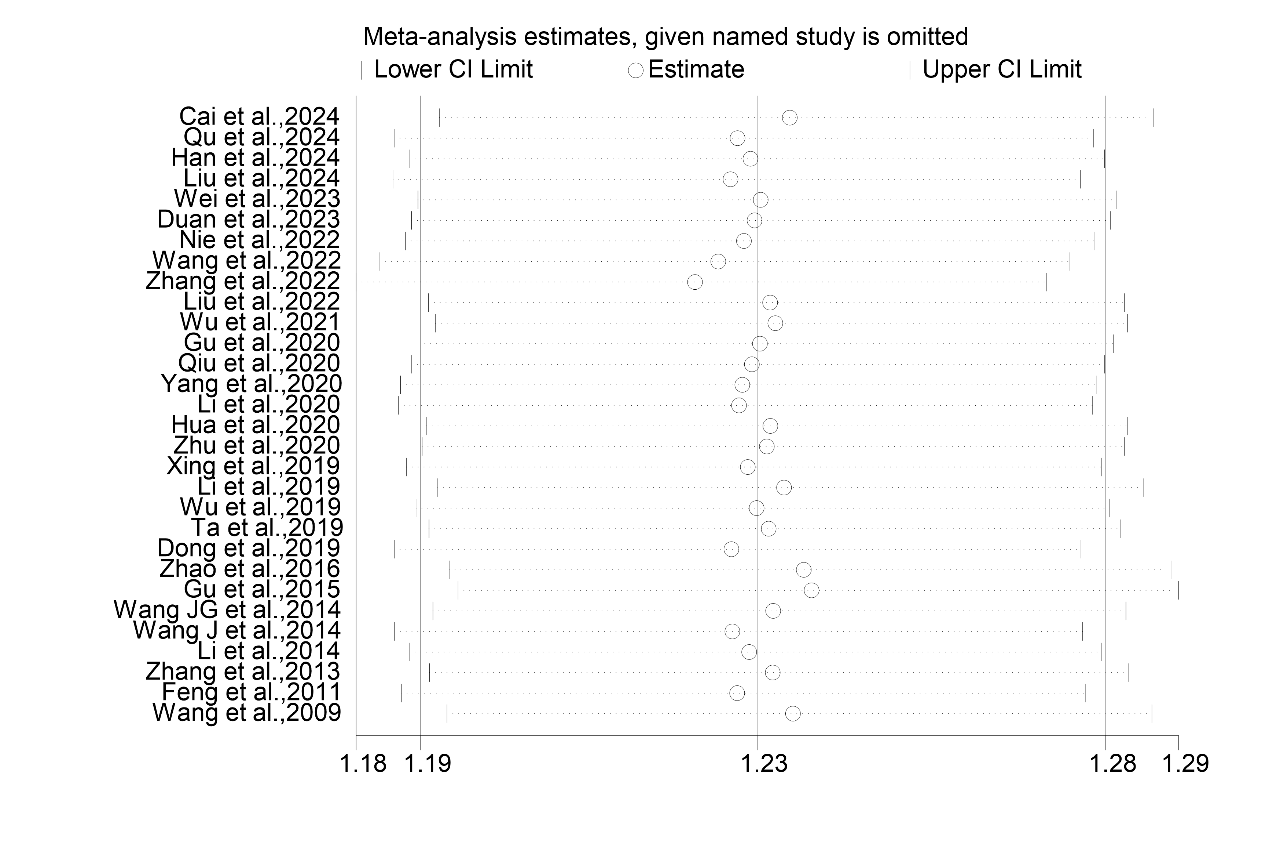


**Figure S3.** Sensitivity analysis results of the total effective rate of acupuncture combined with related rehabilitation therapy in the treatment of PSUI


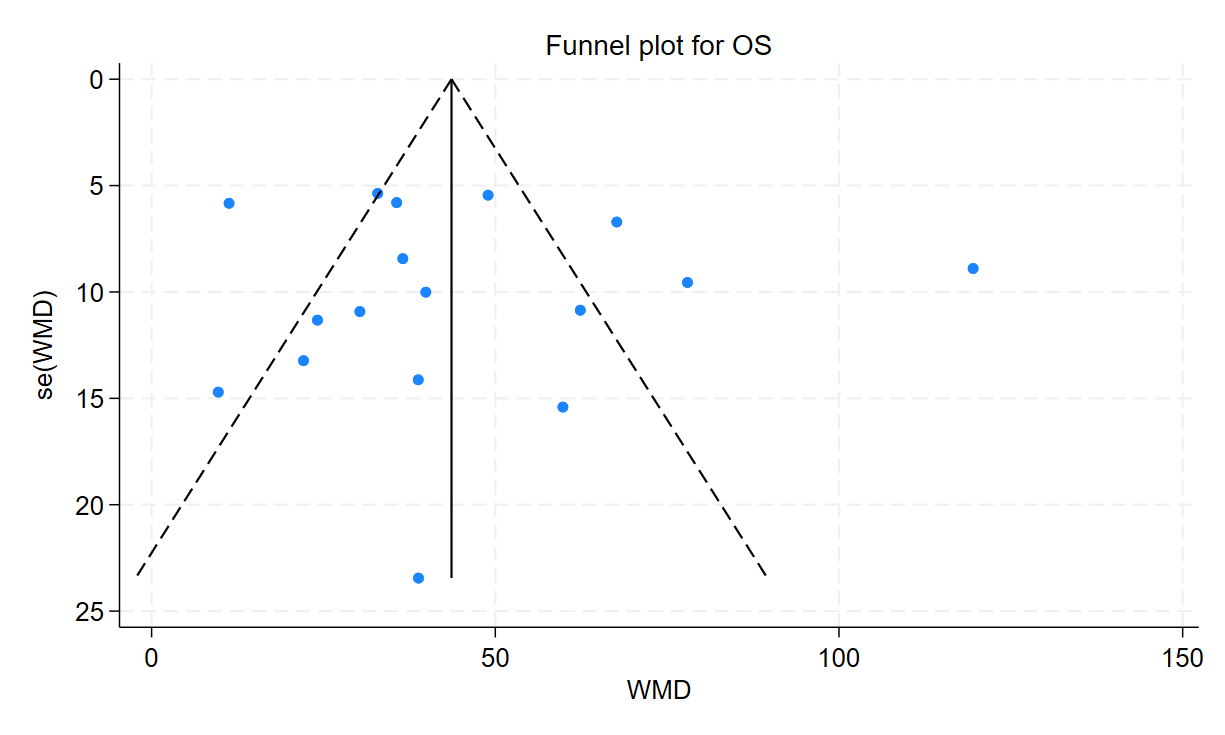


**Figure S4.** Funnel plot of maximum bladder volume of acupuncture combined with related rehabilitation therapy in PSUI


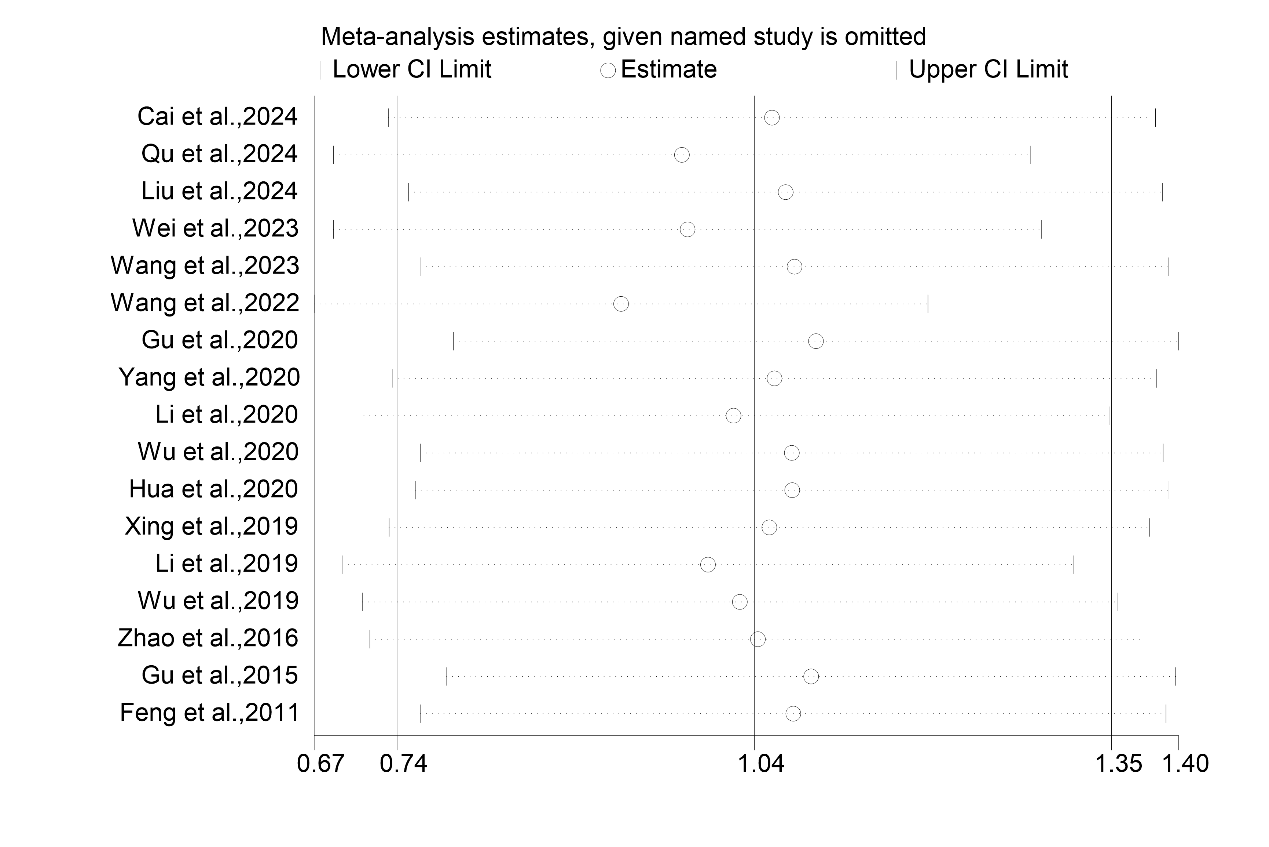


**Figure S5.** Sensitivity analysis results of acupuncture combined with related rehabilitation therapy for the maximum bladder volume of PSUI


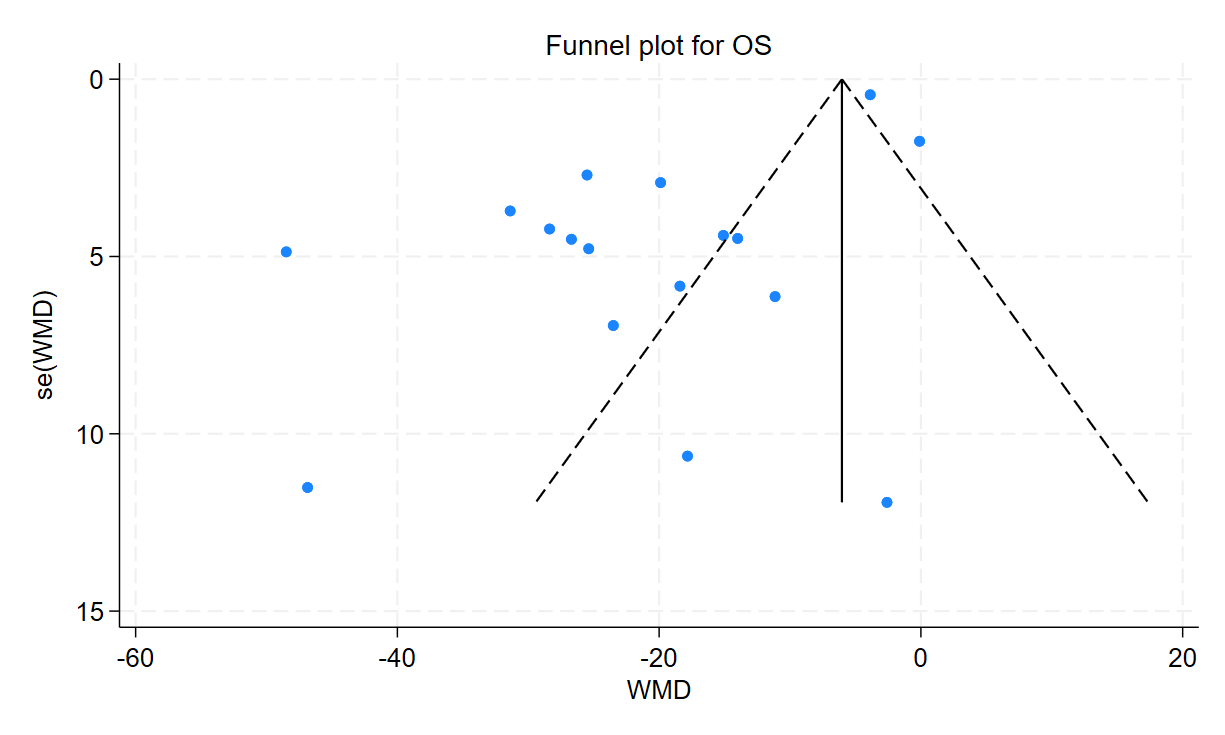


**Figure S6.** Funnel plot of acupuncture combined with related rehabilitation therapy for residual urine volume in PSUI


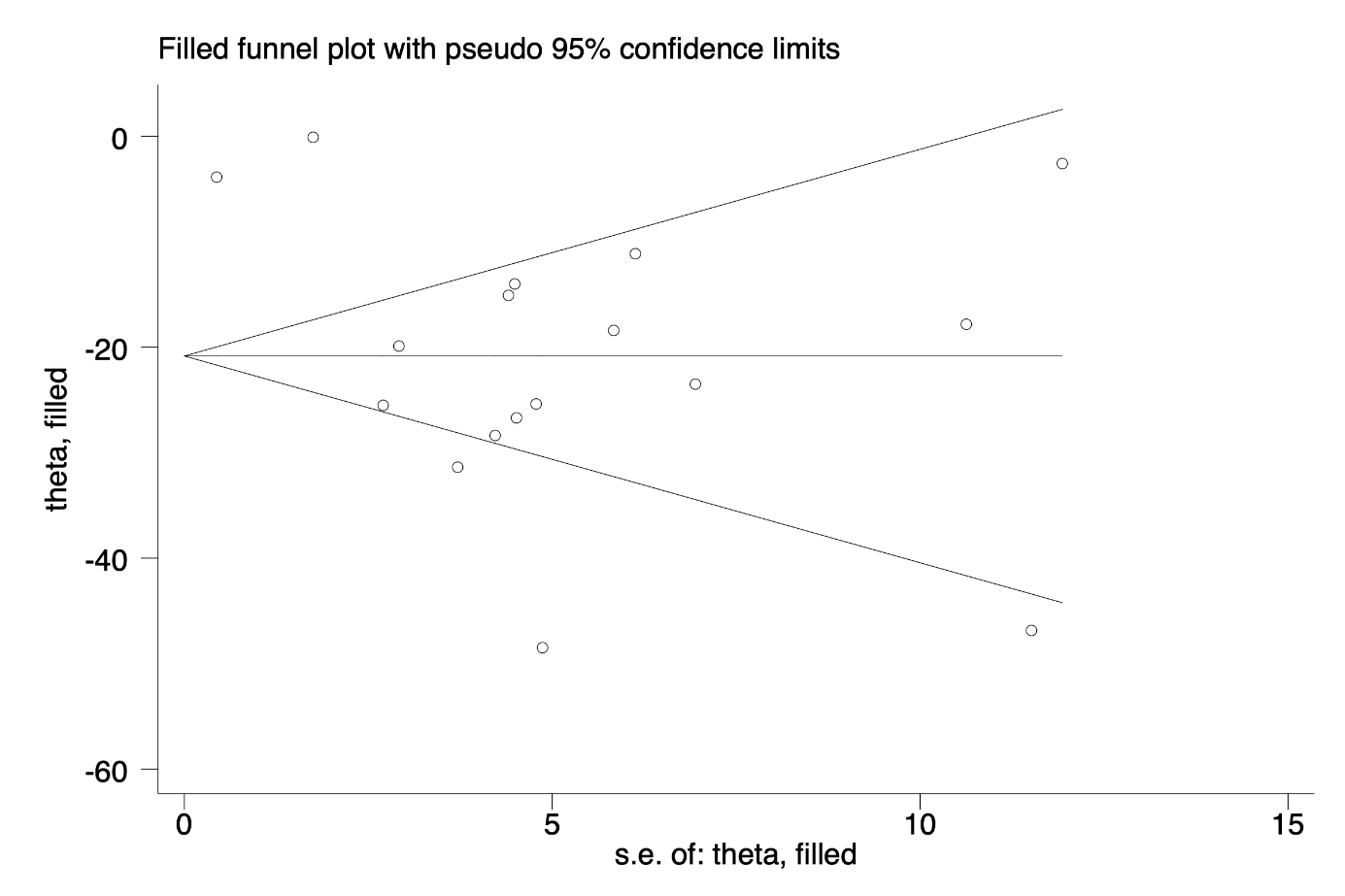


**Figure S7.** The results of the trim and fill method for acupuncture combined with related rehabilitation therapy for residual urine volume in PSUI


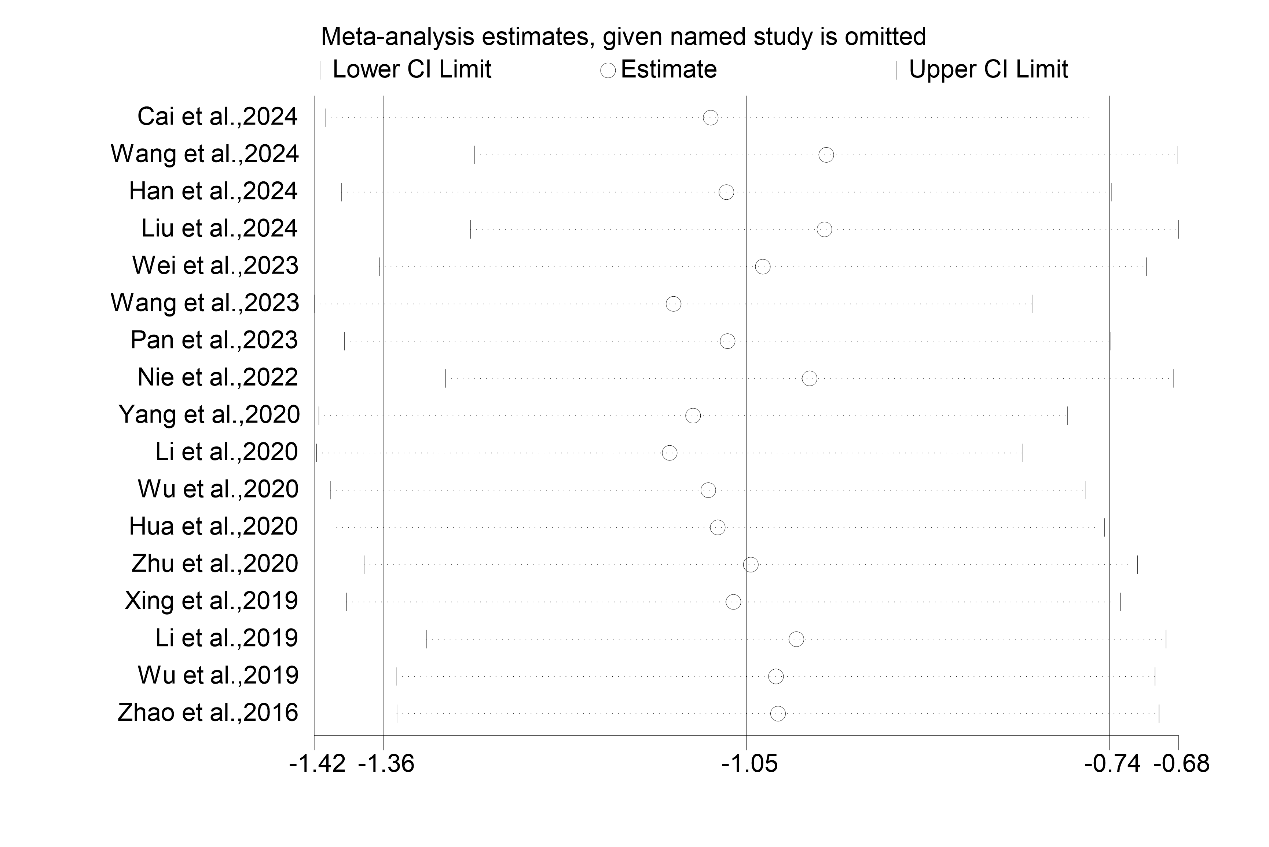


**Figure S8.** Sensitivity analysis results of acupuncture combined with related rehabilitation therapy for residual urine volume in PSUI


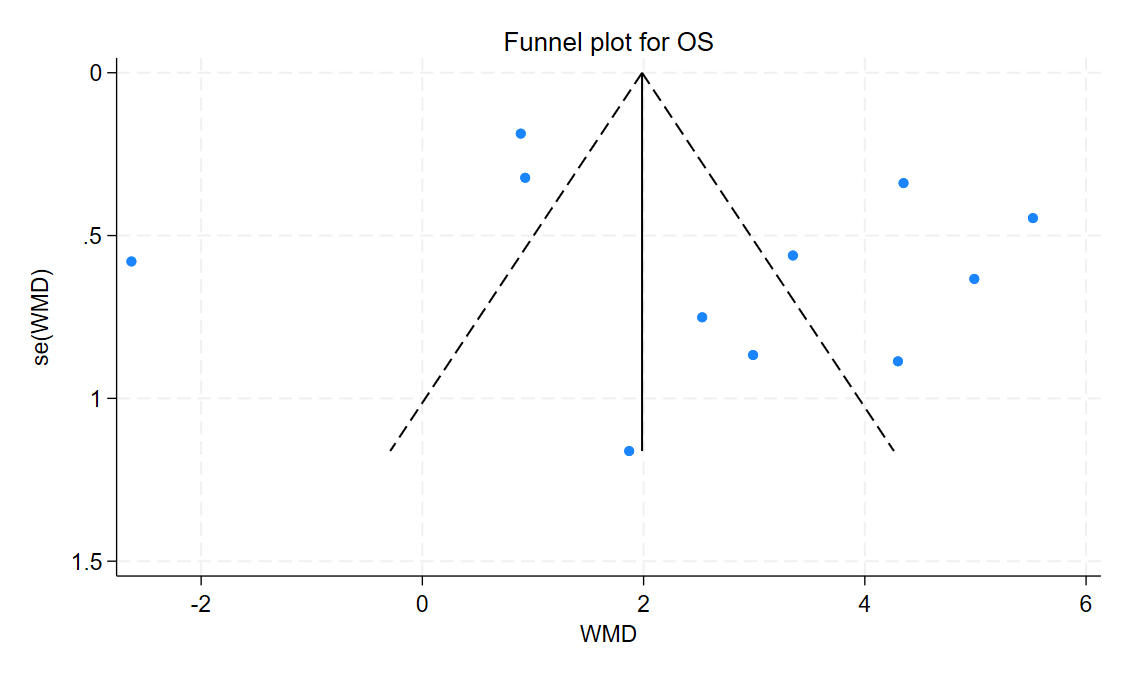


**Figure S9.** Funnel plot of acupuncture combined with related rehabilitation therapy for maximum urine flow rate in PSUI


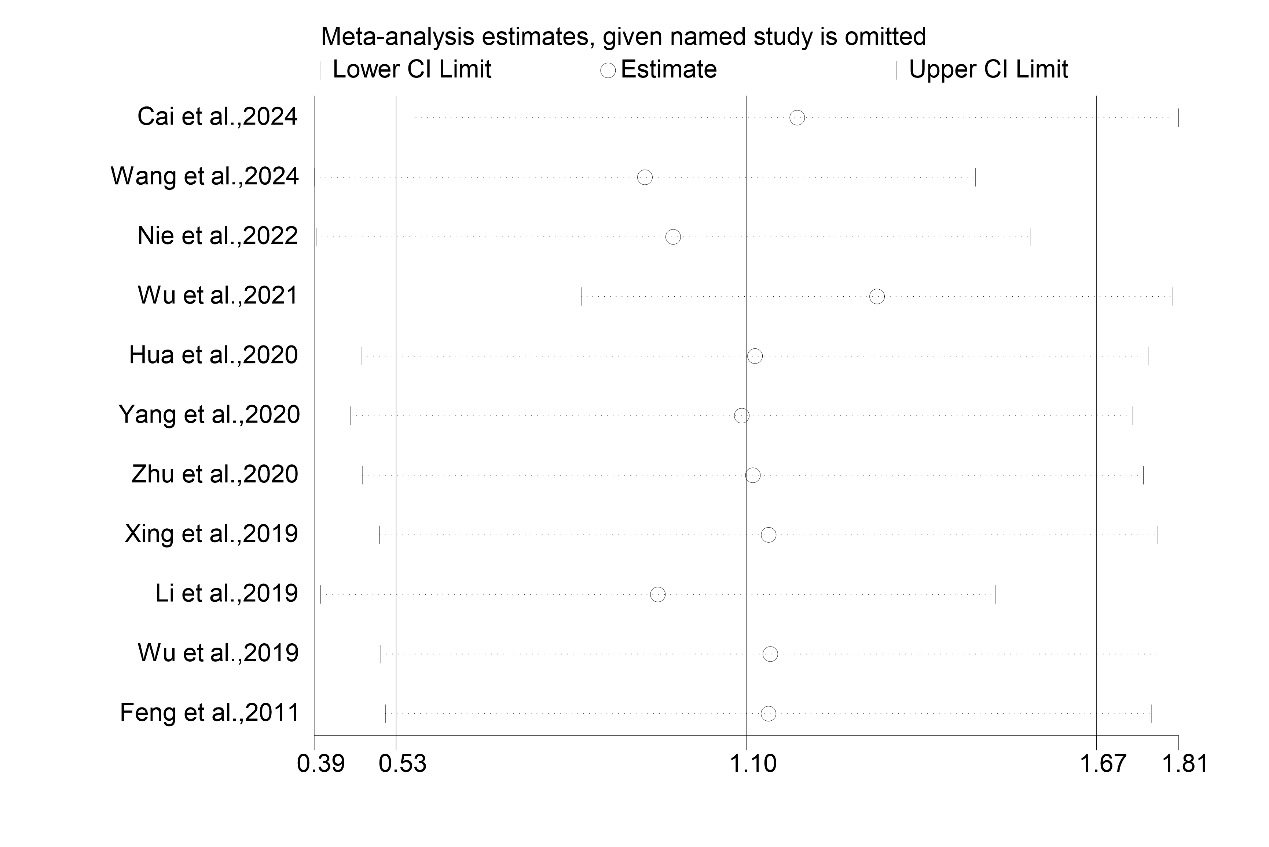


**Figure S10.** Sensitivity analysis results of acupuncture combined with related rehabilitation therapy for maximum urinary flow rate in PSUI


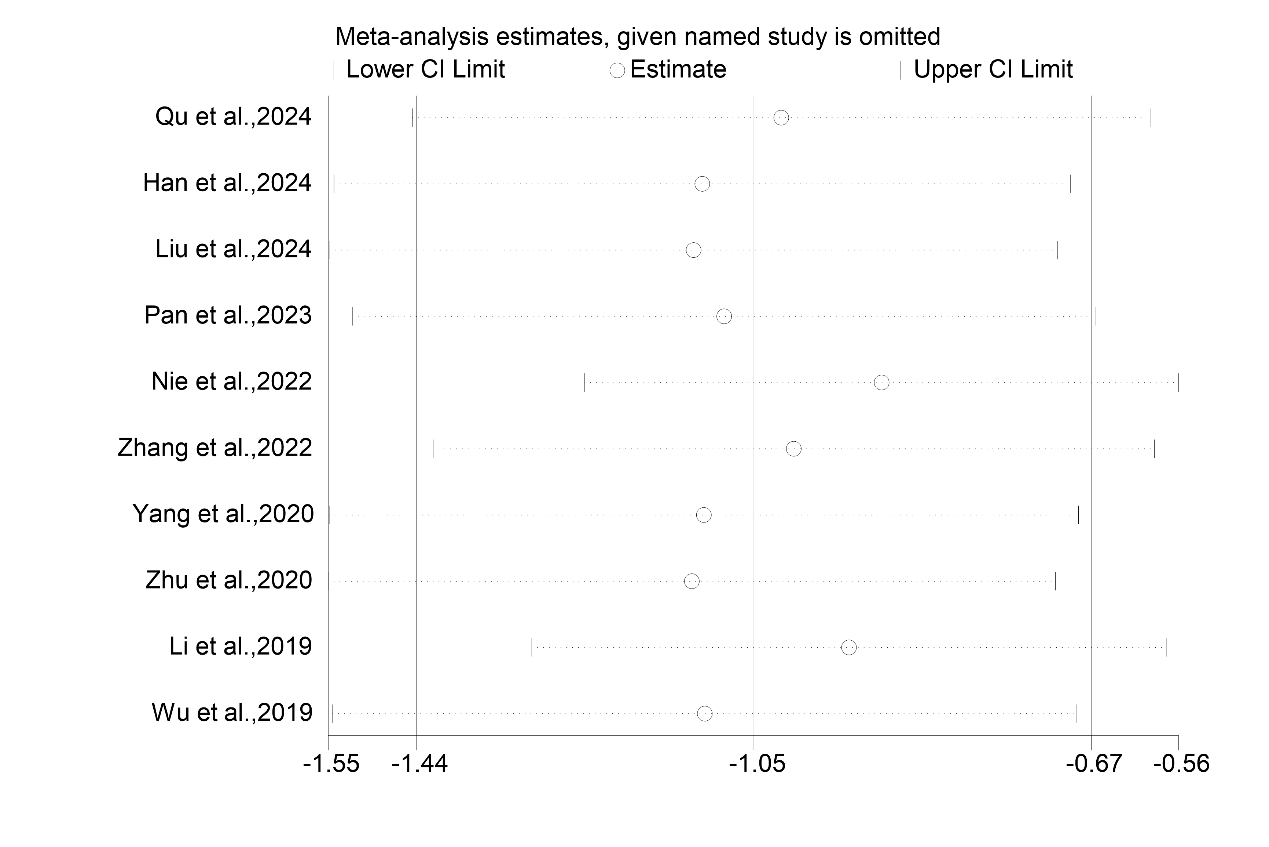


**Figure S11.** Sensitivity analysis results of acupuncture combined with related rehabilitation therapy for the humber of incontinence cases within 24h in PSUI


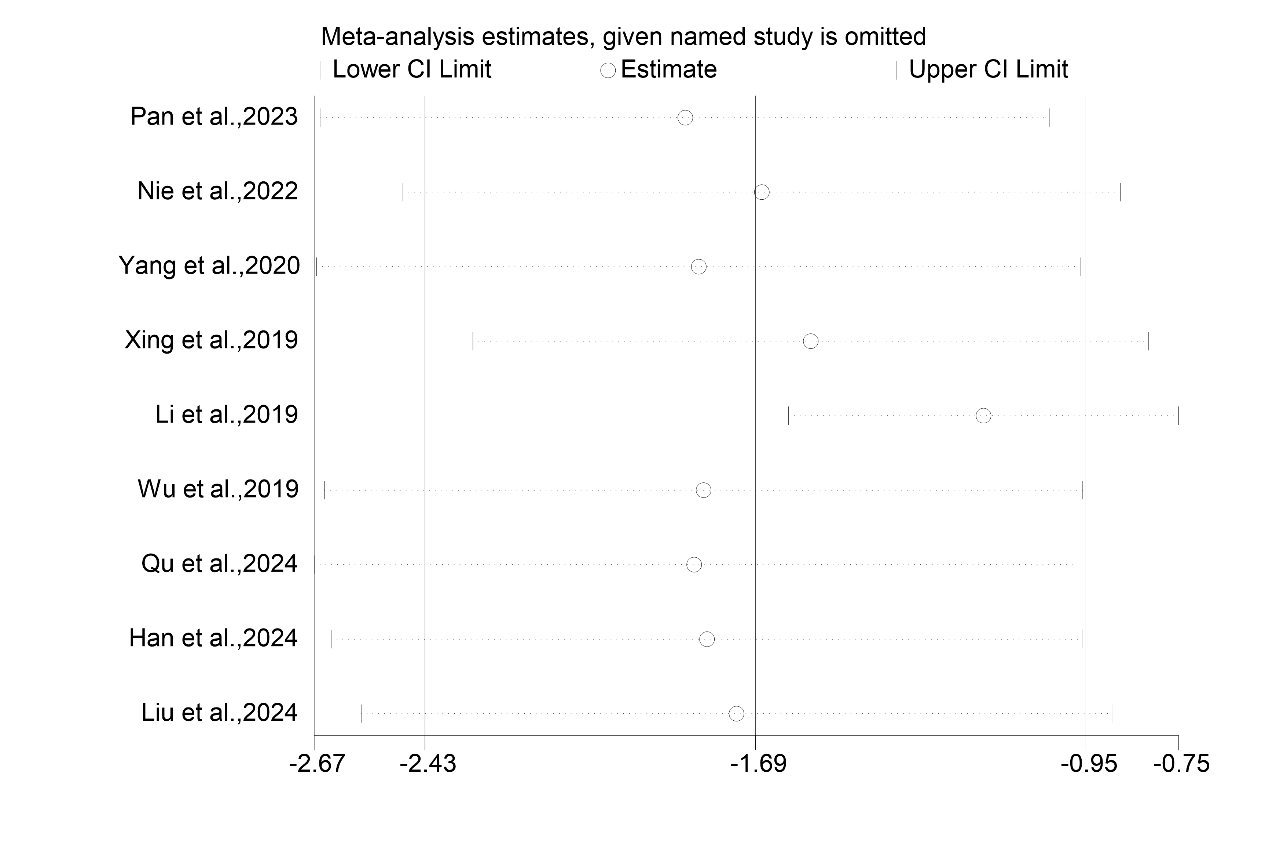


**Figure S12.** Sensitivity analysis results of acupuncture combined with related rehabilitation therapy for the umber of urination within 24h in PSUI


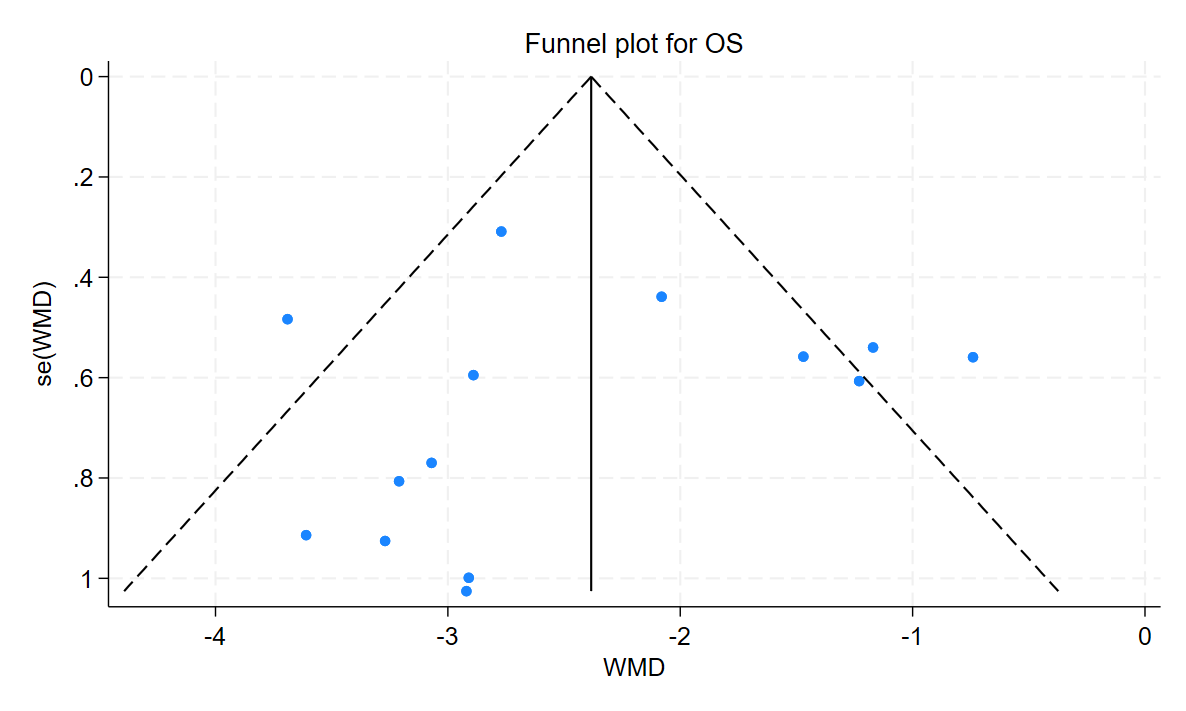


**Figure S13.** Funnel plot of ICIQSF score of PSUI patients treated with acupuncture combined with related rehabilitation therapy


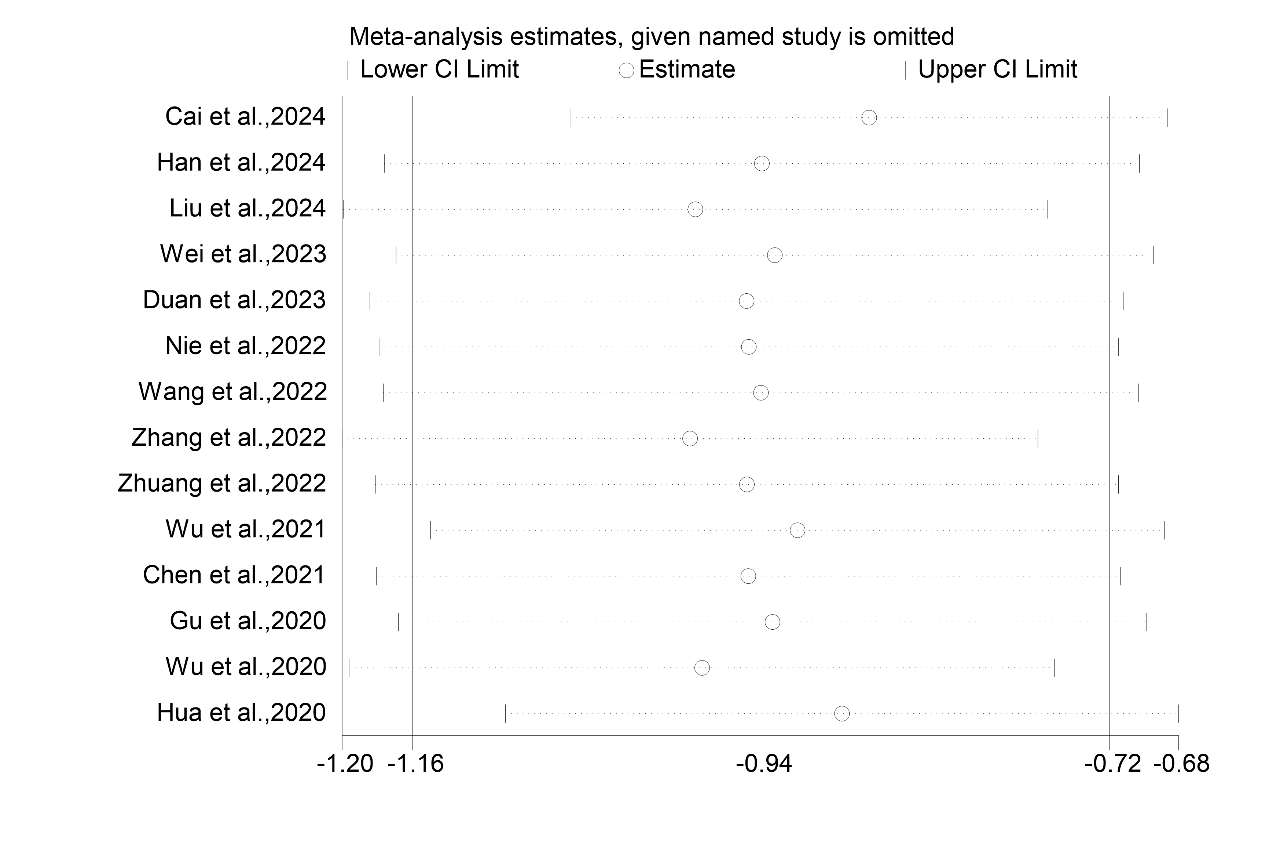


**Figure S14.** Sensitivity analysis results of ICIQSF score of PSUI patients treated with acupuncture combined with related rehabilitation therapy
